# Factors associated with attitudes towards intimate partner violence against women: a comparative analysis of 17 sub-Saharan countries

**DOI:** 10.1186/1472-698X-9-14

**Published:** 2009-07-20

**Authors:** Olalekan A Uthman, Stephen Lawoko, Tahereh Moradi

**Affiliations:** 1 Department of Public Health Sciences, Division of Social Medicine, Karolinska Institutet, Stockholm, Sweden; 2 Department of Public Health & Biostatistics, University of Birmingham, Birmingham, UK; 3 Center for Evidence-Based Global Health, Ilorin, PO Box 5146, Kwara State, Nigeria; 4 Department of Environmental Medicine, Division of Epidemiology, Karolinska Institutet, Stockholm, Sweden

## Abstract

**Background:**

Violence against women, especially by intimate partners, is a serious public health problem that is associated with physical, reproductive and mental health consequences. Even though most societies proscribe violence against women, the reality is that violations against women's rights are often sanctioned under the garb of cultural practices and norms, or through misinterpretation of religious tenets.

**Methods:**

We utilised data from 17 Demographic and Health Surveys (DHS) conducted between 2003 and 2007 in sub-Saharan Africa to assess the net effects of socio-demographic factors on men's and women's attitudes toward intimate partner violence against women (IPVAW) using multiple logistic regression models estimated by likelihood ratio test.

**Results:**

IPVAW was widely accepted under certain circumstances by men and women in all the countries studied. Women were more likely to justify IPVAW than men. "Neglecting the children" was the most common reason agreed to by both women and men for justifying IPVAW followed by "going out without informing husband" and "arguing back with the husband". Increasing wealth status, education attainment, urbanization, access to media, and joint decision making were associated with decreased odds of justifying IPVAW in most countries.

**Conclusion:**

In most Sub-Saharan African countries studied where IPVAW is widely accepted as a response to women's transgressing gender norms, men find less justification for the practice than do women. The present study suggests that proactive efforts are needed to change these norms, such as promotion of higher education and socio-demographic development. The magnitude and direction of factors associated with attitudes towards IPVAW varies widely across the countries, thus suggesting the significance of capitalizing on need-adapted interventions tailored to fit conditions in each country.

## Background

Intimate partner violence against women (IPVAW) is deep-rooted in many African societies, where it is considered a prerogative of men [1,2] and a purely domestic matter in the society [3,4]. IPVAW is one of the greatest barriers to ending the subordination of women. Women, for fear of violence, are unable to refuse sex or negotiate safer sexual practices, thus increasing their vulnerability to HIV if their husband is unfaithful [5,6]. Violence against women, especially by intimate partners, is a serious public health problem that is associated with physical, reproductive and mental health consequences [7-10]. Even though most societies proscribe violence against women, the reality is that violations against women's rights are often sanctioned under the garb of cultural practices and norms, or through misinterpretation of religious tenets. Moreover, when violation takes place within the home, as it is often the case, the abuse is effectively ignored by the tacit silence and the passivity displayed by the state and the law-enforcing machinery. The global dimensions of this violence are alarming as highlighted by numerous studies [2,7,8,11-25].

A troubling aspect of IPVAW is its benign social and cultural acceptance of physical chastisement of women and isthe husband's right to "correct" an erring wife [26]. Women's susceptibility to IPVAW has been shown to be greatest in societies where the use of violence in many situations is a socially accepted norm[27]. Studies have shown that attitude towards IPVAW is one of the most prominent predictors of IPVAW, when contrasted with other potential predictors including social and empowerment factors [28-30]. As it has been emphasised by a number of scholars [31-33], without a fundamental change in the social attitudes that foster, condone, and perpetuate IPVAW we will not be able to respond effectively to this problem, by substantially reducing its alarming rates. Women's own condemnation of this behaviour may, therefore, be an important element in changing it. Most of the studies in the low- and middle-income countries on IPVAW have focused on actual prevalence of IPVAW and its determinants [2,7,8,11-25] and less focus has been on the underlying attitudes towards IPVAW [5,34-38]. In addition, most of the existing studies on IPVAW are based on women's responses while men's perspective may also play an important role. Knowing the extent and reasons for justification of IPVAW in a particular setting is important for different reasons[35]. First, unfettered social and cultural acceptance of IPVAW may not only lead to abetting such practices, but may also create major obstacles toward altering such practices. Hence, understanding the underlying factors related to positive attitude towards IPVAW may be fundamental for designing effective programmes to address the issue. Second, acceptance of IPVAW can be considered as an indicator of the status of women in a specific social and cultural setting. Levels of acceptance of IPVAW can provide insights into the stage of social, cultural and behavioural transformation of a specific society in its evolution towards a more gender egalitarian society.

### Conceptual framework and hypotheses

Building largely upon conceptual framework developed by Rani and colleagues [35], we postulated that an important 'trigger' for IPVAW in patriarchal societies is the transgression of established gender roles. Studies from different patriarchal societies have identified a common set of role expectations for women including preparing food properly, caring for children, seeking husband's or other family member's permission before going out, not arguing with husband, and meeting the sexual needs of the husband [5,35-38]. The conceptual framework used in this study is based on the social learning theory and the ecological framework in order to understand the predictors of attitude towards IPVAW. Social learning theory postulates that individuals learn how to behave by observing and re-enacting the behaviour of role models. Social norms and gender roles in a patriarchal society are learned within a social group and transmitted from generation to generation. The myth of male superiority is maintained in many societies through rigid gender norms and social practices such as polygamy, restriction on movement of women, bride price and other practices that result in overall lower achievement levels among women including education, employment, financial power, public role. Factors that will promote intolerant attitudes towards IPVAW will operate mainly via three mechanisms [35]: by producing a conflict between reality and myth of male superiority; by exposing people to more egalitarian social networks and authority structures other than kin-based ones; and by exposing to non-conformist ideas through modern media. Wealth defines class, which may be characterised by different social networks. Since poverty may increase chances of conflict over resources, it is likely that individuals growing up in poor households and neighbourhoods are often exposed to violence both within and outside the family resulting in high acceptance of violence to resolve conflicts. Furthermore, education and urbanisation may have a greater inverse effect on acceptance of IPVAW among women than among men. The purpose of this study was to contribute to the growing empirical literature on attitudes towards IPVAW. The specific objectives were two-folded; 1) to study gender differences in men's and women's attitudes towards IPVAW and 2) to examine factors associated with attitudes towards IPVAW.

## Methods

### Data

This study used data from Demographic and Health Surveys (DHS) conducted between 2003 and 2007 in sub-Saharan Africa available as of November 2008. DHS surveys were implemented by respective national institutions and ORC Macro International Inc. with financial support from the US Agency for International Development. Methods and data collection procedures have been published elsewhere [39]. Briefly, DHS data are nationally representative, cross-sectional, household sample surveys with large sample sizes, typically between 5,000 and 15,000 households. The sampling design typically involves selecting and interviewing separately nationally representative probability samples of women aged 15–49 years and men aged 15–59 years based on multi-stage cluster sampling, using strata for rural and urban areas and for different regions of the countries. A standardized questionnaire was administered by interviewers to participants in each country. The survey's questionnaireswere similar across countries yielding inter-country comparable data. Only countries with available data on attitudes towards IPVAW were included in this study. This resulted in inclusion of the following 17 participating countries in DHS: Benin, Burkina Faso, Ethiopia, Ghana, Kenya, Lesotho, Liberia, Madagascar, Malawi, Mozambique, Namibia, Nigeria, Rwanda, Swaziland, Tanzania, Uganda and Zimbabwe.

### Outcome variable

To assess the degree of acceptance of IPVAW by women and men, respondents were asked the following question: "Sometimes a husband is annoyed or angered by things which his wife does. In your opinion, is a husband justified in hitting or beating his wife in the following situations?" The five scenarios presented to the respondents for their opinions were: 1. "if wife burns the food," 2. "if wife argues with the husband," 3. "if wife goes out without informing the husband," 4. "if wife neglects the children," and 5. "if the wife refuses to have sexual relations with the husband". Information was collected from all women and men irrespective of their marital status. A binary outcome variable was created for acceptance of IPVAW, coded as '0' if the respondent did not agree with any of the situations when a husband is justified in beating the wife or did not have any opinion on the issue and coded as '1' if the respondent agreed with at least one situation where the husband is justified in beating the wife.

### Determinants variables

To assure consistency, we selected determinant variables based upon previous studies that investigated factors associated with attitudes towards IPVAW.

*Demographic/social position *was assessed using the following indicators: Sex of respondent was defined as men or women; age (15–24, 25–24, 35+ years), place of residence (urban or rural area), occupation (working or not working), education (no education, primary, secondary or higher), marital status (never-, currently-, or formerly married). DHS did not collect direct information on household income and expenditure. We used DHS wealth index as a proxy indicator for socioeconomic position. The methods used in calculating DHS wealth index have been described elsewhere [40-42]. Briefly, an index of economic status for each household was constructed using principal components analysis based on the following households' variables: number of rooms per house, ownership of car, motorcycle, bicycle, fridge, television and telephone, and kind of heating device. From this the DHS wealth index quintiles (poorest, poor, middle, rich, and richest) were calculated and used in the subsequent modelling.

*Media access *was assessed using the following indicators: access to information measured via frequency of watching television, listening to radio, and reading newspapers/magazine. To allow meaningful statistical analysis, we dichotomized the response levels "less than one week", "at least once a week", and "almost every day" as one group and the response level "not at all" as the other group.

#### Decision making power

Respondents' decision autonomy were assessed by inquiring about who bore the responsibility of making decisions on household purchases including small and large ones, visiting relatives and friends, spending the wife's earnings, and the number of children to have. For these variables, response options were "husband," "wife," or "both husband and wife". We created set of additive scale (from 0 to 5) that counted the number of domains in which each (husband/partner alone, wife alone, and couple) had the final word.

### Statistical analyses

In the descriptive statistics the distribution of respondents by the key variables were expressed as percentages. We used Pearson's chi-squared test for analyzing contingency tables. All cases in the DHS data were given weights to adjust for differences in probability of selection and to adjust for non-response. Individual weights were used for descriptive statistics in this study. We used multiple logistic regressions to examine factors associated with attitudes towards IPVAW. We entered all covariates simultaneously in the multiple regression models. Results were presented in the form of odds ratio (ORs) with significance levels and 99% confidence intervals (99% CIs). We performed random-effects estimates models as described by DerSimonian and Laird [43] to incorporate between-country heterogeneity in addition to sampling variation for the calculation of summary OR estimates and corresponding 99% CIs. Between countries heterogeneity was assessed using the Cochran Q test [44] and the *I*^2 ^statistic [45], which describes the percentage of total variation across countries that is the result of heterogeneity rather than chance.*I*^2 ^was calculated based on the formula *I*^2 ^= 100% × (Q - degree of freedom)/Q.

Regression diagnostics were used to judge the goodness-of-fit of the model. They included the tolerance test for multicollinearity, its reciprocal variance inflation factors (VIF), presence of outliers and estimates of adjusted R square of the regression model.

The largest VIF greater than 10 or the mean VIF greater than 6 represent acceptable fit of the models [46,47]. Statistical methods for complex survey data, Stata, release 10.0 (Stata Corp., College Station, TX, USA) were used to account for stratification, clustered sampling and weighing to estimate efficient regression coefficients and robust standard errors. All tests were two tailed. Since due to the large sample size, small differences in attitudes between groups may easily reach the conventional 0.05 statistical significance, we reduced the condition for significance to 0.01 to account for this effect.

### Ethical consideration

This study is based on an analysis of existing survey data with all identifier information removed. The survey was approved by the Ethics Committee of the ORC Macro at Calverton in the USA and by the National Ethics Committee in the respective country. All study participants gave informed consent before participation and all information was collected confidentially.

## Results

### Description of included countries

Table 1 shows the countries, years of data collection, and sample sizes. It also illustrates the demographic and economic diversity of the selected countries. All the 17 countries were low-income countries. As for gross domestic product (GDP) per capita, Swaziland and Namibia emerged as the most affluent countries with values higher than United States dollar (US$)2000 per capita, whilst by contrast Ethiopia, Malawi and Rwanda were the most deprived with values less than US$250 per capita. Nigeria was the most and Lesotho was the least populated country among the countries studied. Regarding levels of urbanization, the percentage of urban population varied across the countries. The percentage of literacy among women was highest in Lesotho (90%) and lowest in Burkina Faso (17%).

**Table 1:** Description of data sets, selected social, economic, and demographic characteristics of the countries included in the study

*Variable*	*year*	*Sample size*		*Population*			*GDP per capita*		Adult literacy rate	
		Men	Women	Total (millions 2005)	Growth rate (1975 – 2005)	%urban (2005)	Value (US$ 2005)	Growth rate (1990 – 2005)	Men	Women
Benin	2006	6000	18000	8.5	3.2	40.1	508	1.4	47.9	23.3
Burkina Faso	2003	3605	12477	13.9	2.8	18.3	391	1.3	31.4	16.6
Ethiopia	2005	6033	14070	79	2.8	16	157	1.5	50.0	22.8
Ghana	2003	5015	5691	22.5	2.6	47.8	485	2.0	66.4	49.8
Kenya	2003	3578	8195	35.6	3.2	20.7	547	-.1	77.7	70.2
Lesotho	2004	2797	7095	2.0	1.8	18.7	808	2.3	73.7	90.3
Liberia	2007	6009	7092	3.4	2.5	58.1	167	2.3	58.3	45.7
Madagascar	2004*	2432	7949	18.6	2.9	26.8	271	-.7	76.5	65.3
Malawi	2004	3261	11698	13.2	3.1	17.2	161	1.0	74.9	54.0
Mozambique	2003	2900	12418	20.5	2.2	34.5	335	4.3	54.8	25.0
Namibia	2007	3915	9804	2.0	2.7	35.1	3016	1.4	86.8	83.5
Nigeria	2003	2346	7620	141.4	2.8	48.2	752	0.8	78.2	60.1
Rwanda	2005	4820	11321	9.2	2.5	19.3	238	0.1	71.4	59.8
Swaziland	2006	4156	4987	1.1	2.5	24.1	2414	0.2	80.9	78.3
Tanzania	2004	2635	10329	38.5	2.9	24.2	316	1.7	77.5	62.2
Uganda	2006	2503	8531	28.9	3.3	12.6	303	3.2	76.8	57.7
Zimbabwe	2006*	7175	8907	13.1	2.5	35.9	259	-2.1	92.7	86.2

(Source: ^†^Demographic and Health Surveys of the respective countries; ^‡^UNDP Human Development Report)

GDP: gross domestic product, HDI: human development index, GDI: gender-related development index

Table 2 shows the socio-demographic characteristics of the study participants. Most of the respondents were female. The percentage of female ranged from 53% in Ghana to 81% in Mozambique. Most of the respondents (34% to 48%) were aged 15–24. The percentage of respondents with no education varies across the country. The percentage of respondents with no education was lowest in Zimbabwe (3%) and highest in Burkina Faso (76%). With the exception of Lesotho (44%), more than 50% of the respondents were currently working. The percentage of respondents that are currently married ranged from 35% in Namibia to 73% in Benin. Respondents were fairly evenly distributed across the wealth status strata. In most countries, most of the respondents were living in the rural areas. Burkina Faso (11%) had least number of respondents with access to newspaper; Swaziland (68%) had the highest. In all countries studied, more than 50% had access to radio. The percentage of respondents with access to television ranged from 17% in Malawi to 59% in Ghana.

**Table 2:** Percentage distribution by selected characteristics

	Benin	Burkina Faso	Ethiopia	Ghana	Kenya	Lesotho	Liberia	Madagascar	Malawi	Mozambique	Namibia	Nigeria	Rwanda	Swaziland	Tanzania	Uganda	Zimbabwe
	
Variable	%	%	%	%	%	%	%	%	%	%	%	%	%	%	%	%	%
**Sex**																	
Men	23.0	22.4	30.0	46.8	30.4	28.3	45.9	23.4	21.8	18.9	28.5	23.5	29.9	45.5	20.3	22.7	44.6
Women	77.0	77.6	70.0	53.2	69.6	71.7	54.1	76.6	78.2	81.1	71.5	76.5	70.1	54.5	79.7	77.3	55.4
**Age**																	
15–24	33.9	40.3	40.7	36.4	42.8	45.3	37.9	36.3	43.2	40.8	41.8	41.2	43.3	48.4	41.6	41.6	46.6
25–34	34.0	27.4	29.3	29.4	29.3	25.3	29.2	30.5	31.6	29.5	30.8	29.2	27.1	26.8	31.5	30.3	28.4
35+	32.1	32.3	29.9	34.2	27.9	29.4	32.8	33.2	25.2	29.6	27.4	29.6	29.7	24.8	26.9	28.1	25.1
**Education**																	
No education	58.8	76.2	54.2	28.3	13.5	07.3	29.8	14.6	20.7	32.0	08.6	35.1	21.2	08.1	22.0	17.4	03.1
Primary	21.9	13.2	24.4	18.4	53.3	58.8	34	39.8	62.6	56.6	27.7	22.8	67.2	33.5	64.0	59.1	31.6
Secondary+	19.2	10.6	21.4	53.3	33.3	33.9	36.2	45.6	16.7	11.3	63.7	42.1	11.6	58.3	14.0	23.5	65.3
**Occupation**																	
Working	80.5	86.7	50.5	79.2	64.9	44.9	71.1	75.2	63.6	72.9	56.9	61.2	67.9	50.7	79.1	88.1	54.6
Not working	19.5	13.3	49.5	20.8	35.1	55.1	28.9	24.8	36.4	27.1	43.1	38.8	32.1	49.3	20.9	11.9	45.4
**Marital**																	
Never married	22.6	24.9	31.3	32.8	34.4	38.0	32.6	26.3	19.7	21.1	58.7	31.9	40.5	56.5	28.2	26.9	36.7
Currently married	73.1	71.6	59.6	60.0	57.2	49.9	59.8	61.9	70.2	66.3	35.0	63.7	49.2	36.6	63.0	61.7	52.8
Formerly married	04.3	03.5	09.1	07.2	8.4	12.1	7.5	11.8	10.2	12.6	06.3	04.4	10.3	06.9	08.8	11.4	10.5
**Wealth**																	
Poorest	18.7	16.7	19.7	23.9	16.3	17.2	19.3	12.5	16.7	18.9	15.7	19.1	19.5	14.9	17.4	20.5	17.8
Poor	19.1	18.5	15.0	18.2	15.8	19.8	19.8	10.7	20.1	15.1	17.4	18.0	18.7	16.4	18.5	19.0	18.5
Middle	19.3	23.1	14.6	17.5	17	18.3	19.8	13.1	21.8	17.6	24.1	19.6	18.1	18.8	18.1	17.5	18.2
Richer	21.0	16.4	14.3	18.6	19.7	20.5	20.7	18.7	21.3	20.7	25.1	20.8	19.6	21.7	22.7	18.3	23.0
Richest	21.8	25.2	36.5	21.8	31.2	24.2	20.3	44.9	20.1	27.6	17.7	22.5	24.1	28.2	23.3	24.7	22.5
**Type of residence**																	
Urban	42.0	24.5	30.1	39.9	33.1	26.7	43.7	64.3	14.3	43.7	44.3	40.6	23.2	32.6	24.0	16.7	35.2
Rural	58.0	75.5	69.9	60.1	66.9	73.3	56.3	35.7	85.7	56.3	55.7	59.4	76.8	67.4	76.0	83.3	64.8
**Decision making indices**																	
Respondent alone (1–5)	58.2	62.6	42.9	59.1	73.6	70.2	53.1	65.2	66.4	73.3	31.2	52.7	62.4	16.2	70.3	45.7	16.8
Husband/Partner alone (1–5)	43.5	57.5	42.9	29.6	52.7	46.0	45.4	23.9	58.5	51.8	30.1	56.8	28.8	16.9	54.0	55.7	10.9
Husband-wife (1–5)	35.1	19.5	58.4	24.6	42.7	38.6	59.9	52.2	42.6	42.0	43.5	29.9	41.1	14.9	36.7	46.8	28.1
**Media access**																	
Read newspaper	14.1	11.0	26.5	28.8	51.1	23.6	32.9	43.7	32.8	19.0	65.6	30.4	29.4	68.2	42.1	28.2	50.3
Listen to radio	84.5	72.6	53.7	89.6	85.6	58.9	70.5	79.1	83.0	86.8	89.1	78.7	81.4	83.6	80.7	82.7	64.3
Watch television	40.8	30.2	32.3	59.0	46.4	18.0	44.1	47.2	17.1	36.7	51.5	49.8	17.1	48.4	33.2	18.3	44.7

### Justification of IPVAW by gender norm transgressed

**Figure 1:**
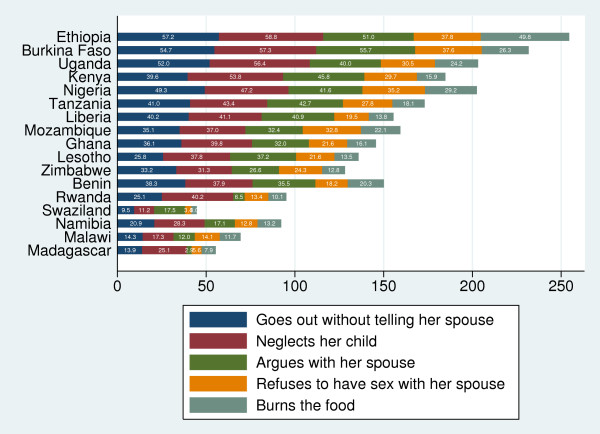
**Percentage of women who believe that IPVAW is justified, by different scenarios**.

**Figure 2:**
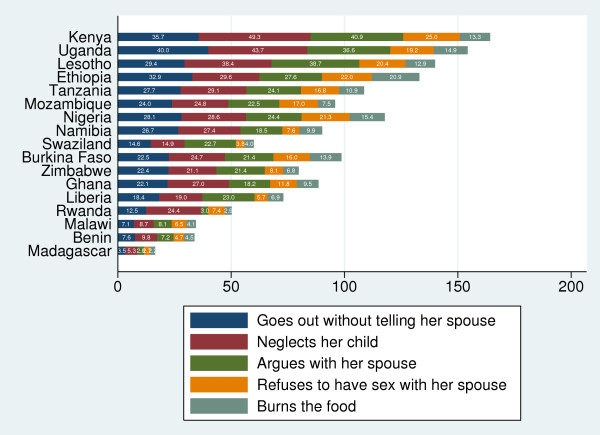
**Percentage of men who believe that IPVAW is justified, by different scenarios**.

**Figure 3:**
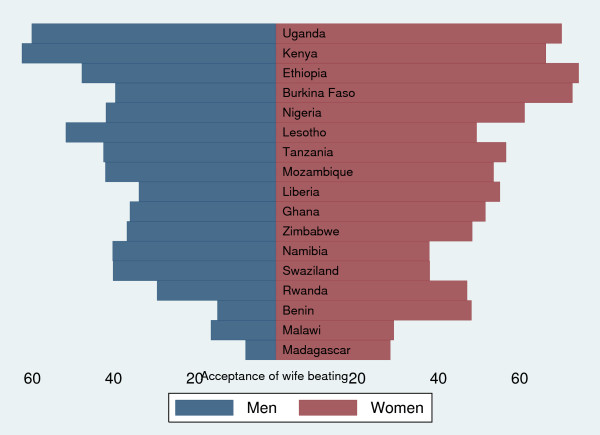
**Sex-difference in attitude toward IPVAW of 17 sub-Saharan countries**.

"Neglecting the children" was the most common reason agreed by both women (Figures 1) and men (Figures 2) for justifying IPVAW followed by going out without informing husband and arguing back with the husband. The proportion of respondents who agreed with the statement that IPVAW is justified for "neglecting the children" ranged from 5% in Madagascar to 49% in Kenya among men and from 11% in Swaziland to 59% in Ethiopia among women. The justification for IPVAW was relatively low for "refusing sexual relations" among scenarios presented. Women were consistently more likely to justify IPVAW than men in all the countries, with the exception of Lesotho, Swaziland and Kenya (Figure 3). The percentage of women who justified IPVAW was lowest in Madagascar (28%) and highest in Ethiopia (74%). Madagascar had also the lowest percentage (8%) of men who justified IPVAW and Kenya the highest (62%).

### Factors associated with attitudes towards IPVAW

Table 3 presents the adjusted OR for justification of IPVAW (see additional file 1 for full odds ratios, 99% CI and p-values). The diagnosis of multi-collinearity is shown in additional file 1. The largest VIF ranged from 2.15 to 5.18; and the average VIF ranged from 1.77 to 2.49. Since none of the VIF values exceeds 10 and none of the average VIF exceeds 6, we concluded that there was no multi-collinearity problem. Women were significantly more likely to justify IPVAW than men in all countries studied with the exception of Lesotho. Women were 29% less likely to justify IPVAW than men in Lesotho (OR = 0.71, 99% CI 0.60 – 0.84). The association between sex and justification of IPVAW became non-significant in Namibia, Kenya, and Swaziland after controlling for respondents' socio-demographic factors, decision making autonomy, and access to media. Compared to respondent aged 35 and older, respondent aged 15–24 were consistently and significantly more likely to justify IPVAW in all countries except for Benin and Burkina Faso. Lower educational attainment was positively associated with acceptance of IPVAW. Respondents with no education or primary education were more likely to justify IPVAW compared with those with secondary or higher education in all countries but Liberia, Madagascar, and Nigeria. Relationship between occupation and acceptance of IPVAW was mixed. Respondents not in working force from Burkina Faso, Mozambique and Rwanda were at 20% statistically increased risk of justifying IPVAW. Currently not working respondents from Benin, Liberia, Madagascar, Malawi, Tanzania, and Zimbabwe were less likely to justify IPVAW. The association was not significant in other seven countries.

**Table 3:** Factors associated with attitudes towards intimate partner violence against women identified by multiple logistic regression analyses*

	*Benin*	*Burkina Faso*	*Ethiopia*	*Ghana*	*Kenya*	*Lesotho*	*Liberia*	*Madagascar*	*Malawi*	*Mozambique*	*Namibia*	*Nigeria*	*Rwanda*	*Swaziland*	*Tanzania*	*Uganda*	*Zimbabwe*
	
Variable	OR	OR	OR	OR	OR	OR	OR	OR	OR	OR	OR	OR	OR	OR	OR	OR	OR
**Sex**																	
Women (vs men)	4.40	3.71	4.03	1.73	ns	0.71	4.37	5.36	1.99	1.38	ns	1.49	2.13	ns	2.01	1.76	1.35
**Age**^†^																	
15–24	ns	ns	1.28	1.77	1.67	1.45	1.49	1.41	1.44	1.26	1.25	1.44	1.38	2.56	1.56	1.34	2.21
25–34	ns	ns	ns	1.48	ns	ns	1.17	ns	ns	1.13	ns	ns	ns	1.24	1.19	ns	1.44
**Education**^†^																	
No education	2.12	2.19	2.20	2.09	2.46	1.61	ns	ns	1.33	1.84	ns	1.36	1.91	1.42	2.04	ns	2.06
Primary	1.44	1.99	1.97	1.69	2.23	1.61	ns	ns	1.44	1.67	1.21	ns	1.85	1.54	2.45	1.31	1.38
**Occupation**^†^																	
Not working	0.86	1.20	ns	ns	ns	ns	0.79	0.78	0.80	1.19	ns	ns	1.20	ns	0.86	ns	0.85
**Marital**^†^																	
Currently married	1.44	ns	ns	ns	1.46	ns	ns	1.35	0.69	ns	0.69	ns	0.81	ns	ns	ns	0.75
Formerly married	1.60	1.42	1.57	ns	1.52	ns	1.40	ns	0.56	ns	ns	ns	0.78	ns	0.78	ns	ns
**Wealth**^†^																	
Poorest	2.65	ns	ns	3.24	1.87	1.77	1.24	1.47	1.47	1.22	5.85	2.08	ns	1.69	1.70	1.42	2.88
Poor	2.14	ns	1.26	2.06	1.78	1.59	1.25	ns	1.54	1.38	3.94	1.83	ns	1.61	1.63	1.73	2.72
Middle	2.21	ns	ns	1.93	1.66	1.31	ns	ns	1.40	1.44	3.08	1.56	1.17	1.36	1.48	1.45	2.25
Richer	1.79	1.23	1.28	1.64	1.73	ns	ns	1.22	1.38	1.43	1.71	1.47	1.18	1.28	1.24	1.44	1.61
**Type of residence**																	
Rural (versus urban)	1.18	ns	2.06	ns	ns	1.22	1.15	0.73	1.21	1.15	ns	ns	1.15	1.38	ns	1.49	1.24
**Decision making indices**																	
Respondent alone (0–5)	0.92	0.95	1.22	ns	1.12	ns	1.38	ns	1.11	0.95	1.12	ns	1.10	ns	1.08	1.08	1.13
Husband/Partner alone (0–5)	1.06	ns	1.21	ns	1.11	1.09	1.36	1.14	1.13	1.12	1.20	1.21	1.11	1.10	ns	1.23	1.15
Husband-wife (0–5)	0.92	0.88	ns	0.91	0.88	0.81	1.18	0.90	0.92	na	na	0.93	ns	ns	0.91	ns	1.08
**Media access**																	
Read newspaper	0.84	0.77	0.79	0.81	0.80	0.82	0.85	ns	1.20	ns	0.81	0.75	0.88	ns	ns	ns	0.74
Listen to radio	ns	ns	ns	ns	ns	ns	ns	0.83	ns	ns	ns	ns	0.80	ns	ns	ns	1.23
Watch television	ns	ns	0.85	0.85	ns	ns	ns	0.81	ns	1.22	1.26	ns	ns	ns	1.18	ns	ns

Abbreviations: OR: odds ratio; ns: not significant; na: not available

*Shown are odds ratio that are significant at p = 0.01

Reference group: Age- 35 or older; Education – secondary or more; occupation – working; marital status – never married; wealth status – richest

Compared with those never married, respondents that were currently married from Benin (OR = 1.44, 99% CI 1.19 – 1.75), Kenya (OR = 1.46, 99% CI 1.21 – 1.77), and Madagascar (OR = 1.35, 99% CI 1.02 – 1.77) were more likely to justify IPVAW. While, those currently married from Malawi, Namibia, Rwanda, and Zimbabwe were less likely to justify IPVAW than those never married. In some countries, such as Benin, Burkina Faso, Ethiopia, Kenya, and Liberia those formerly married were more likely to justify IPVAW. In other countries, such Malawi (OR = 0.56, 99% CI 0.43 – 0.72), Rwanda (OR = 0.78, 99% CI 0.64 – 0.96), and Tanzania (OR = 0.78, 99% CI 0.63 – 0.97) those formerly married were less likely to justify IPVAW than never married. The odds of justifying IPVAW increased with decreasing wealth status in all countries. Living in rural areas increased the odds of justifying IPVAW in most of the countries. However, those living in rural areas in Madagascar (OR = 0.73, 99% CI 0.62 – 0.86) were less likely to justify IPVAW than their counterparts from urban areas. Association of justifying IPVAW with decision making indices were not consistent across the countries studied. Respondents who reported final say in more household decisions than their partners were more likely to justify IPVAW in nine countries and less likely to justify IPVAW in Benin, Burkina Faso, and Mozambique. Respondents were more likely to justify IPVAW in most countries when their partners alone had the final say in more household decisions than they did. When respondents reported more decisions being made jointly than individually, they were significantly less likely to justify IPVAW in most countries.

Access to newspaper reduced the odds of justifying IPVAW in all countries with the exception of Malawi (OR = 1.20, 99% CI 1.06 – 1.37). The association between listening to radio and acceptance of IPVAW was significant in only three countries. As expected, listening to radio reduced the odds of justifying IPVAW in Madagascar (OR = 0.83, 99% CI 0.70 – 0.99) and Rwanda (OR = 0.80, 99% CI 0.72 – 0.90). Counter intuitively, access to radio increased the likelihood of justifying IPVAW in Zimbabwe (OR = 1.23, 99% CI 1.10 – 1.37). The association between watching television and odds of justifying IPVAW was not consistent across countries. In some countries, such as Ethiopia, Ghana, and Madagascar watching television reduced the likelihood of justifying IPVAW. In other countries, such as Mozambique, Namibia and Tanzania watching television increased the odds of justifying IPVAW.

**Figure 4:**
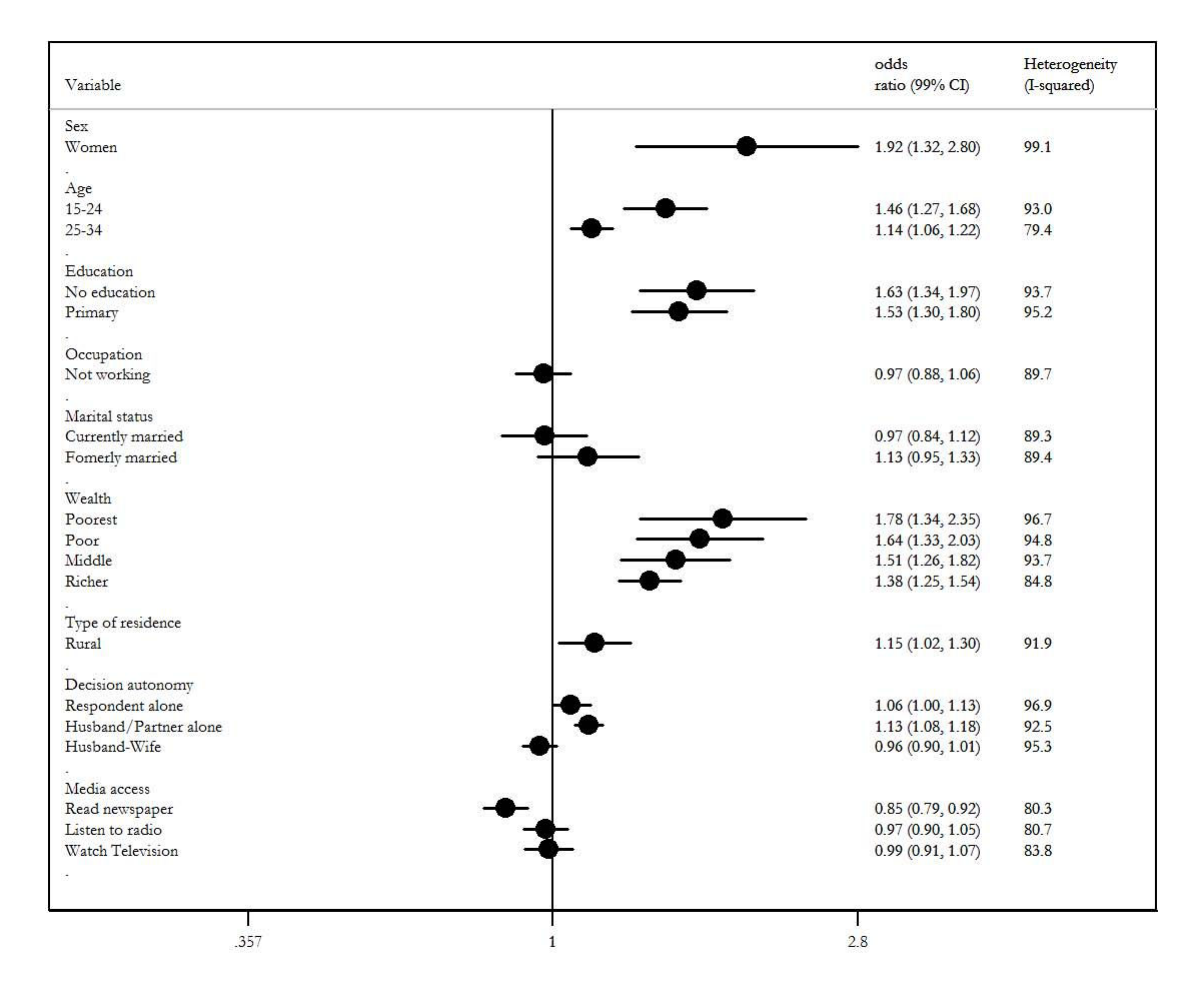
**Forest plot showing pooled odds ratio and 99% confidence for socio-demographic factors**.

Figure 4 shows the results of pooled odds ratios (weight average) of the determinants of attitudes towards IPVAW (see additional file 2 for forest plots for each variable). The results of meta-analyses confirmed that sex, age, education attainment, wealth status, when partner alone had the final say in household decisions, and access to newspaper were associated with attitudes towards IPVAW in the pooled analyses. Random effect model meta-analysis showed that women were more likely to justify IPVAW than men (pooled OR = 1.98, 99% CI 1.32 to 2.80). The results from the pooled analyses also confirmed that odds of justifying IPVAW increase with decreasing age, decreasing education attainment, decreasing wealth status. Compared those living in the urban areas, those from rural were more likely to justify IPVAW (pooled OR = 1.15, 99% CI 1.02 to 1.30). Random effect model meta-analysis showed respondent were more likely to justify IPVAW when their partners alone had the final say in more household decision that they did (pooled weighted average OR = 1.13, 99% CI 1.08 to 1.18). The pooled OR for the effect of access to newspaper was 0.85 (99% 0.79 to 0.92). The results of pooled analyses for occupation, marital status, when respondents reported more final say in more, when respondents reported more decisions being made jointly, access to radio and television were not significant. Figure 4 also shows magnitude of cross countries variability in the determinants of attitudes towards IPVAW. The Cochran Q's test for heterogeneity for all variables gave p-values which were highly significant (p < .0001). Higgins and Thompson statistics suggested that 79% to 99% of the total variation in the estimated effect of determinants was due to heterogeneity between countries, thus suggesting that between countries heterogeneity were almost certain present.

## Discussion

In this large comparative study from 17 countries in sub-Saharan Africa, we found that IPVAW was widely acceptable under certain circumstances and more such among women, younger people, less educated, poorest, those living in rural areas, those with less access to media and single decision makers. Women were more likely to justify IPVAW in all countries, with exception of Lesotho even after controlling for confounding factors. This, in agreement with the result of previous study that has examined this association in seven countries in sub-Saharan Africa conducted between 1999 and 2001[35]. A possible explanation for the exception seen in Lesotho could be due to the fact that the adult female literacy rate is higher than adult male literacy rate. It has been reported that women are more vulnerable to abuse and exploitation in environment where there is high gender inequality, these factors may be responsible for the observed gender disparities in attitudes toward IPVAW. More sophisticated measures such as decomposition analyses are needed to explore the sources of gender disparities in attitudes towards IPVAW.

Evidence from meta-analyses suggests that sex of the respondent stood out as the most important predictor of attitudes towards IPVAW. Results of meta-analyses provided evidence that wealth status and education attainment were also significantly associated with attitudes IPVAW. Access to newspaper reduced likelihood of having tolerant attitudes towards IPVAW. Some of the socio-demographic correlates we studied have been documented in literature [12-20]. We found that younger people are more likely to justify IPVAW. Despite the cross-sectional design in this study, comparing trend in attitude by ages indicates that the younger generation is more likely to accept IPVAW than the older one. However, there is a need for longitudinal studies to confirm this finding. Wealth status, education and urbanisation had a greater negative impact on acceptance of IPVAW in most countries in this study. The limited effects seen in primary education alone compared to those with secondary or higher education is not surprising. Having few years of education usually at young age may not expose people to new non-conformists ideas[35]. It may even bring conflict between reality and myth of male superiority[35]. In accordance with the results of previous studies[5,35], we found that occupation status had minimal effect on acceptance of IPVAW. Most women in low-income countries work largely in informal sectors with low paid jobs. Women are usually exposed to the same patriarchal social structures at the work place that may further strengthen the myth of male superiority.

### Policy implications

We have provided evidence that in most Sub-Saharan African countries studied here IPVAW is widely accepted as a response to women's transgressing gender norms, men find less justification for the practice than do women. We believe like others [35,48], that the first step toward eliminating this practice is to "build up a substantial amount of momentum" in opposition to the use of violence in conflict resolution and that, given its widespread acceptance in these societies, the development of a "new social consensus" albeit a slow process, is crucial. A climate of tolerance of IPVAW would make it easier for perpetrators to persist in their violent behaviour and make it more difficult for women to disclose domestic violence [31]. In terms of IPVAW, there is a need for a social environment characterized by low tolerance and an increased sense of social and personal responsibility toward IPVAW [33]. This, in turn, would contribute to a social environment more effective in terms of social control of IPVAW [49]. Public awareness and education campaigns aiming to lowering social tolerance and to increase the sense of social and personal responsibility toward IPVAW are needed in order to reduce and prevent IPVAW.

Sub-Saharan Africa is ethnically, culturally and religiously diverse and economic development and education levels vary widely across the countries. Not unexpectedly, we found that the magnitude and directions of factors associated with attitudes towards IPVAW varies widely across the 17 countries studied. Sub-Saharan African countries have heterogeneous conditions. Understanding cross countries diversities may aid in the identification of regions that may need to be particularly targeted with education and prevention programs. Thus, multifaceted geographically differentiated intervention may represent a potentially effective approach for addressing issues related to intimate partner violence in sub-Saharan Africa with policies tailored to country-specific conditions. Furthermore, decision makers should capitalize on need-adapted interventions to meet societal conditions in a bid to change men's distorted attitudes toward IPVAW.

Potential public health programmes could include structural and gender-based interventions. Structural interventions focusing on improving the coverage and dissemination of information to the general public may be beneficial in changing men's attitudes toward IPVAW, alongside a review of the educational system, which may seem to reinforce gender inequity. It is also important to note that access to media reduced odds of acceptance of IPVAW in most countries. The widespread acceptance of IPVAW may also become a major hurdle in success of other reproductive health programs (i.e., family planning programs), care seeking for sexually transmitted diseases or voluntary testing and counselling, and condom use for prevention of HIV/AIDS if the women do not confront men because of the threat of domestic violence, as a large proportion of women in these societies considered "arguing with husband" and "refusing sex" as valid reasons for wife beating[50]. Gender-based interventions, building on advocacy for shared autonomy in the domestic domain, and the provision of basal education for all may prove paramount in changing men's distorted attitudes about IPVAW, particularly among younger men and in rural settings. We found that joint decision making reduced likelihood of justifying IPVAW indicating that imbalance of power is associated with higher odds of justifying IPVAW. Interventions that promote joint decision-making might be a promising strategy for increasing women's view towards equality in marriage while promoting men's views that household disputes should be settled with negotiation and not violence. To break the norms that sustain women's vulnerability in society, there is a need for pro-active efforts toward socioeconomic development and promotion of higher education.

### Study limitations and strengths

There are a number of caveats to be considered when interpreting these results. The cross-sectional nature of the data limits ability to draw casual inferences. The study can be criticized for using an indirect measure of household wealth. However, due to the fact that in low- and middle-income countries, it is hard to obtain reliable income and expenditure data, an asset-based index is generally considered a good proxy for household wealth status. Our study focused on understanding the role of individual variables as determinants of attitudes towards violence in specific Sub-Saharan countries. We did not incorporate an assessment of the effect of interactions between such variables and other societal factors in our study design. For instance societal level variables such as ethnicity could interact with gender in explaining attitudes towards violence. Future research using a multi-level design may be necessary to assess such interaction effects. Another important limitation is that the reliability and validity of this instrument used for measuring attitudes towards IPVAW is yet to be established [36,37]. It has been documented that attitudes toward IPVAW is limited in scope to capture women's normative roles in the domestic arena[37]. In addition, other issues such as motivations for partner abuse because of nondomestic factors such as women's financial status, employment position, education and husband's drunkenness are not included in the measure of attitudes toward IPVAW. Apart from instrumental validity, the potential limitations of face-to-face interviews need to be acknowledged[37]. For example, when contrasted with self administered questionnaires, participants may tend to underreport their attitudes toward IPVAW in the presence of their interviewers. However, ethical measures such as guarantees of anonymity and administering the interviews by trained personal may have improved such reporting[37].

Despite these limitations, the study strengths are significant. It is a large, population-based study with national coverage. In addition, data of the DHS are widely perceived to be of high quality, as they were based on sound sampling methodology with high response rate. DHS also adhere to stringent ethical rules in the collection of domestic violence data used. An important strength of this study is the number of included countries and geographic and socioeconomic diversities constitute a good yardstick for the region, and help to strengthen the findings from the study.

## Conclusion

This large comparative analysis has provided evidence that IPVAW was widely acceptable under certain circumstances and more such among women, younger people, less educated, poorest, those living in rural areas, those with less access to media and single decision makers. There is a need for proactive efforts to break the norms that sustain women's vulnerability in the society besides socio-economic development as well as promotion of higher education among men and women. Direct concerted efforts from the government, non-governmental organisations and enlightened men and women within the society are necessary to raise awareness about the issue as well as questioning the social norms. This study has provided information about individual predictors of attitudes toward IPVAW in 17 sub-Saharan countries. However, our knowledge about the contextual factors associated with the attitudes toward IPVAW is still limited.

## Competing interests

The authors declare that they have no competing interests.

## Authors' contributions

OAU, SL and TM were involved in the conception of the study. OAU carried out data extraction. OAU conducted statistical analysis under supervision of SL and TM. OAU drafted the paper with contributions from the co-authors. All authors read and approved the final manuscript.

## Pre-publication history

The pre-publication history for this paper can be accessed here:

http://www.biomedcentral.com/1472-698X/9/14/prepub

## Supplementary Material

Additional file 1 Multiple logistic regression results showing adjusted odds ratio (with 99% confidence interval and p-values) for relationship between acceptance of intimate partner violence against women and different determinant variables among men and women in sub-Saharan Africa. Click here for file

Additional file 2 Forest plot of the odds ratios (ORs) and 99% confidence intervals (CIs) of individual countries and pooled data for socio-demographic factors. Click here for file
