# Supplementary material for: Factors associated with attitudes towards intimate partner violence against women: a comparative analysis of 17 sub-Saharan countries
Source: BMC Int Health Hum Rights. 2009 Jul 20;9:14. doi: 10.1186/1472-698X-9-14 (PMC2718859; doi:10.1186/1472-698X-9-14)

Forest plot of the odds ratios (ORs) and 99% confidence intervals (CIs) of individual countries and pooled data for sex of the respondents

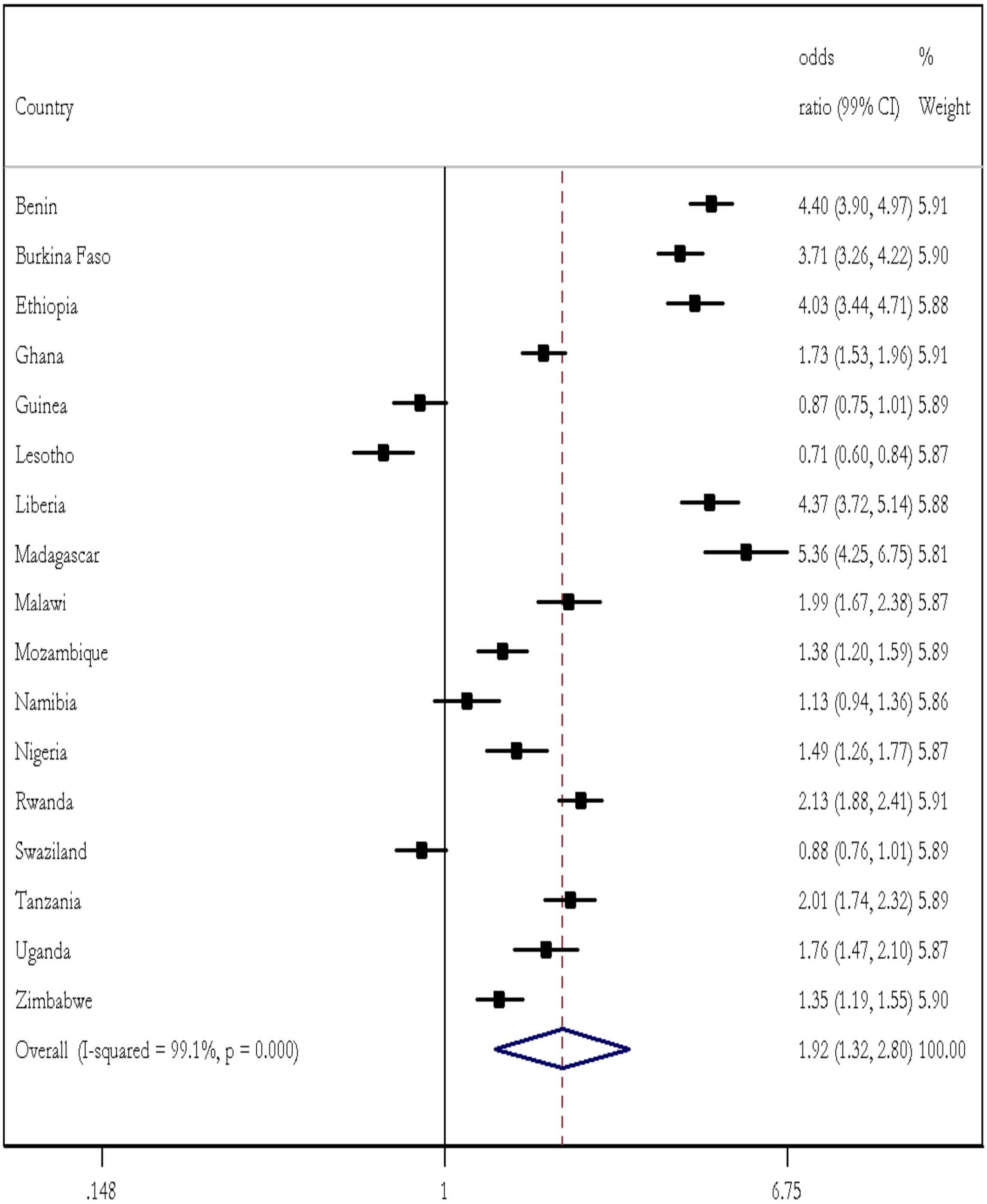

Forest plot of the odds ratios (ORs) and 99% confidence intervals (CIs) of individual countries and pooled data for Age group (15 – 25 versus 35 or older)

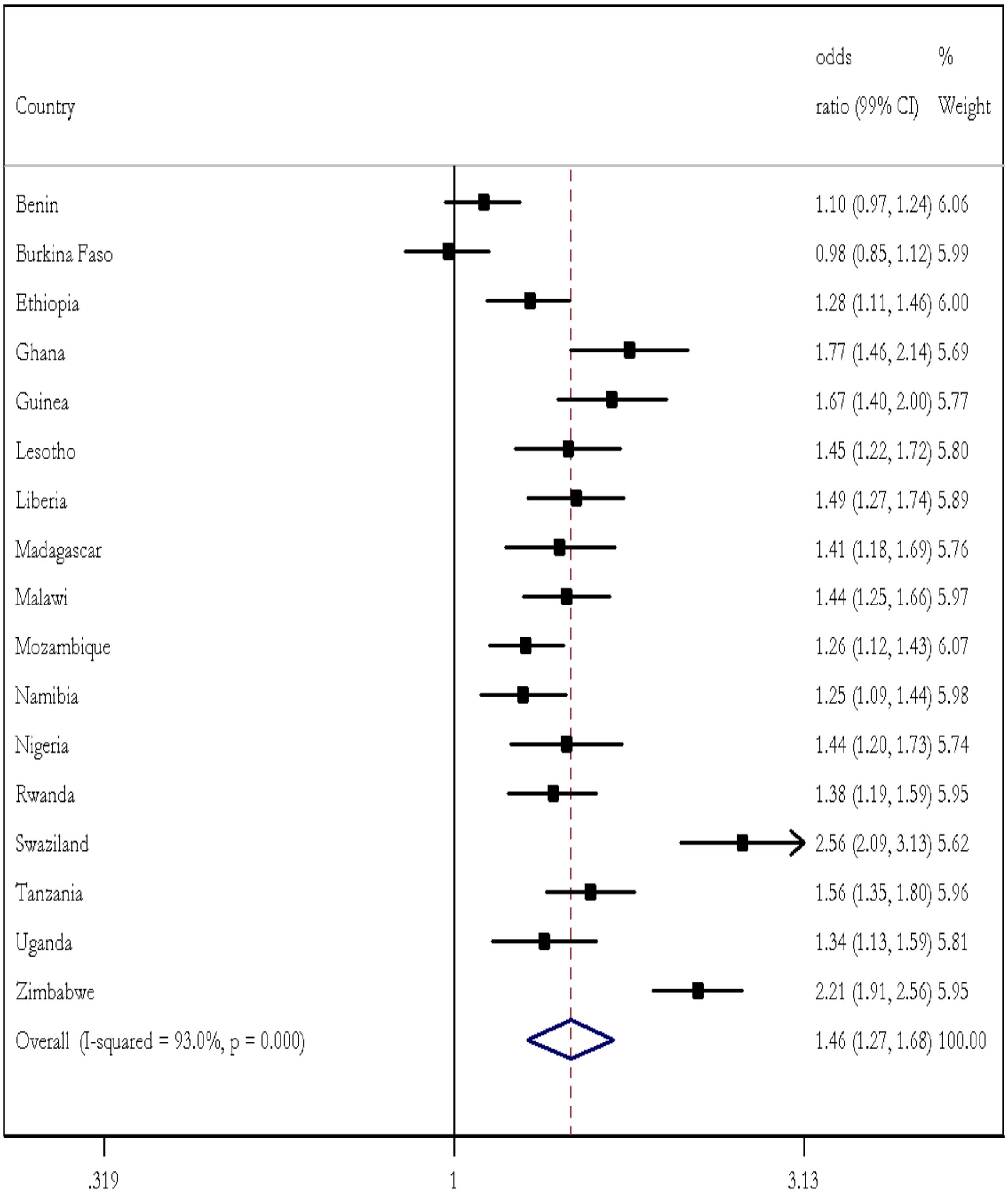

Forest plot of the odds ratios (ORs) and 99% confidence intervals (CIs) of individual countries and pooled data for Age group (25-34 versus 35 or older)

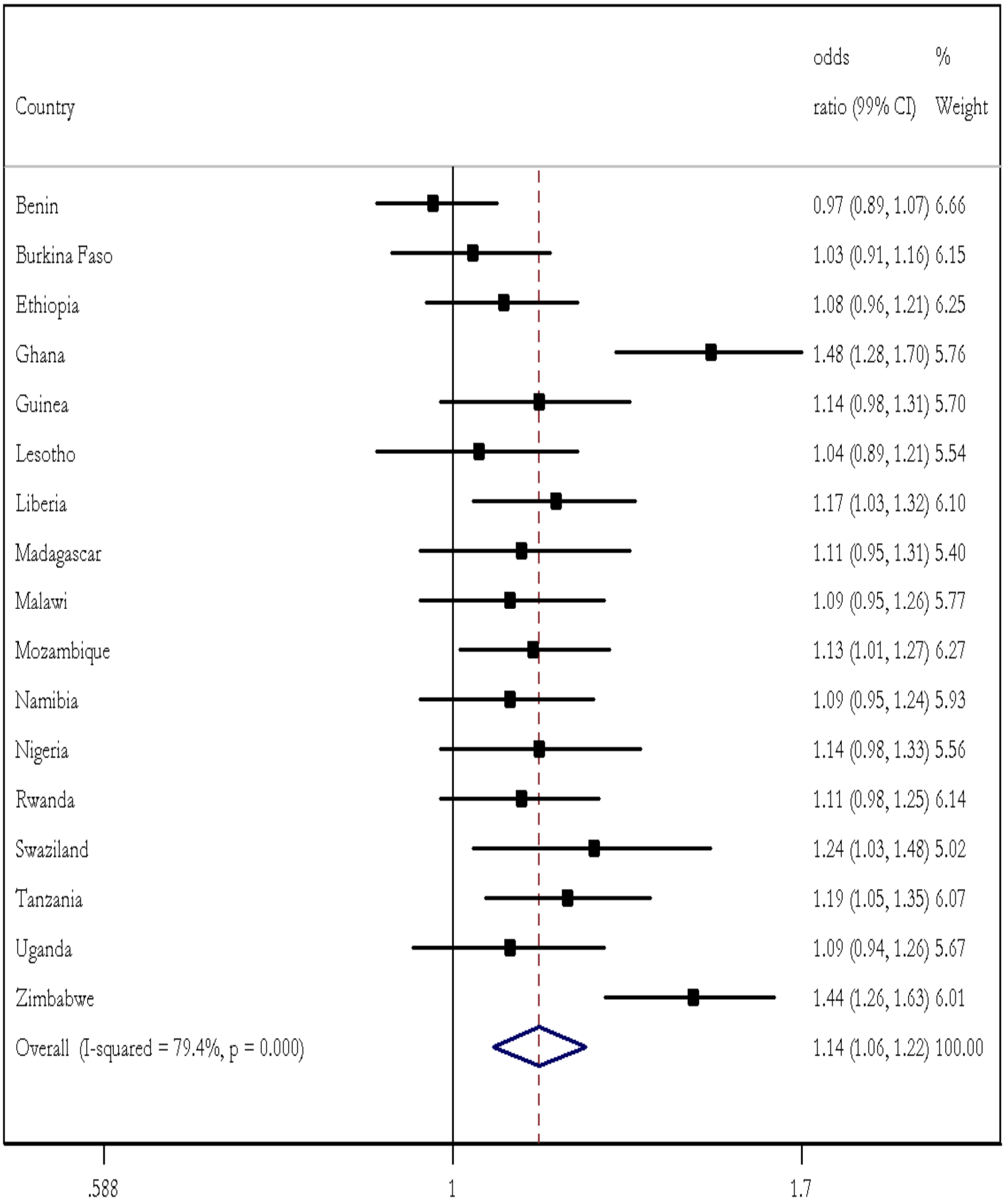

Forest plot of the odds ratios (ORs) and 99% confidence intervals (CIs) of individual countries and pooled data for education attainment (no education)

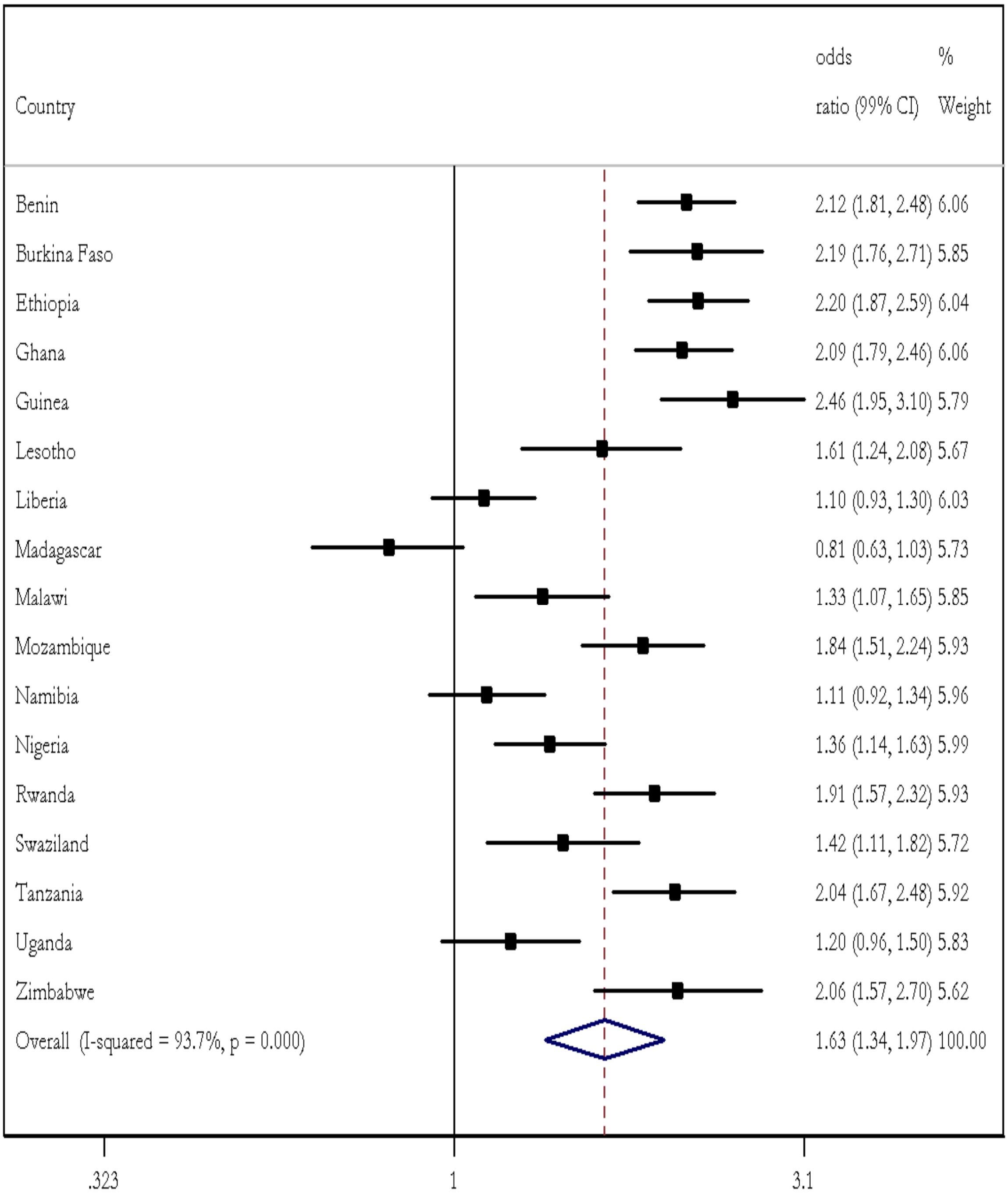

Forest plot of the odds ratios (ORs) and 99% confidence intervals (CIs) of individual countries and pooled data for education attainment (Primary)

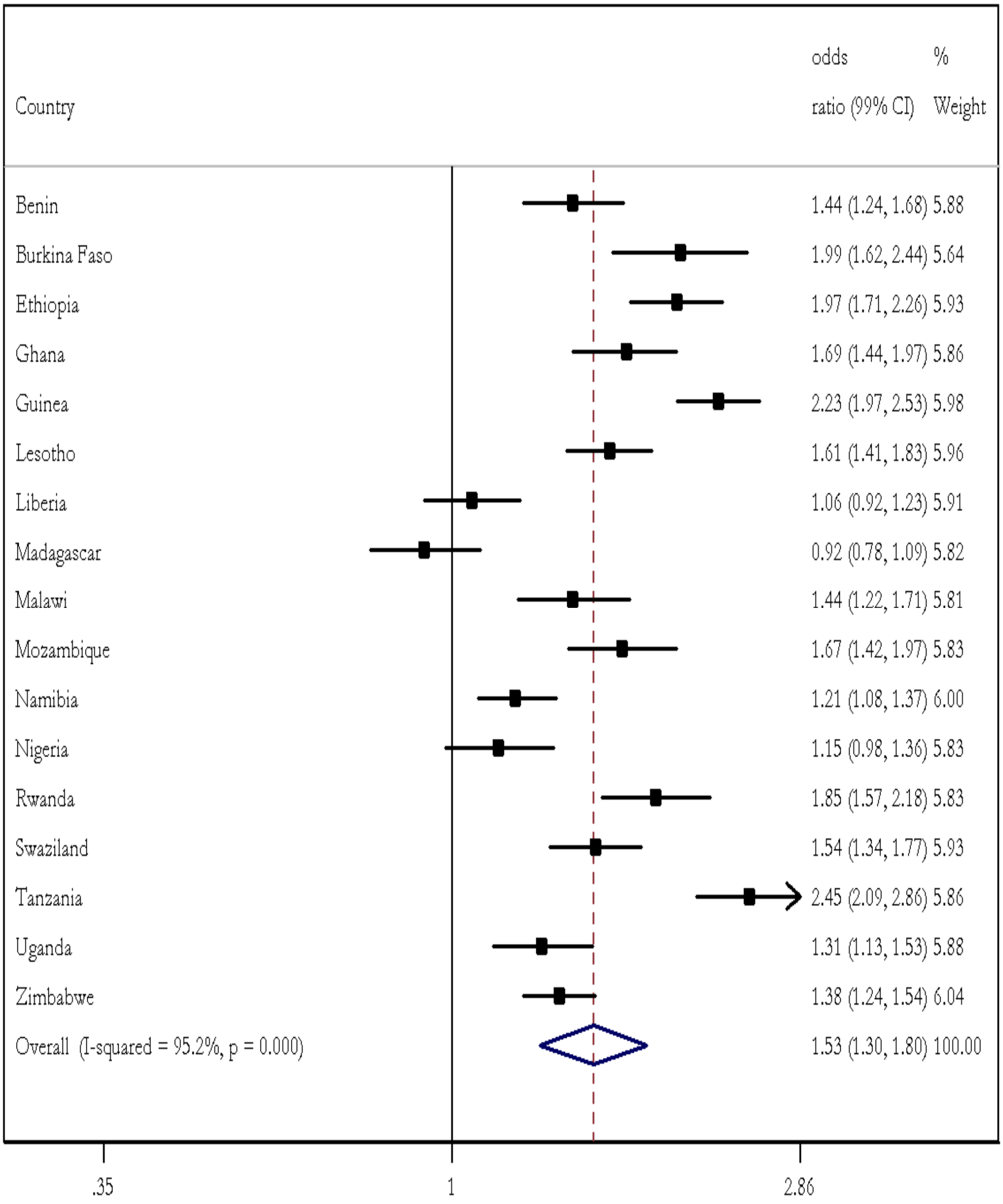

Forest plot of the odds ratios (ORs) and 99% confidence intervals (CIs) of individual countries and pooled data for occupation status

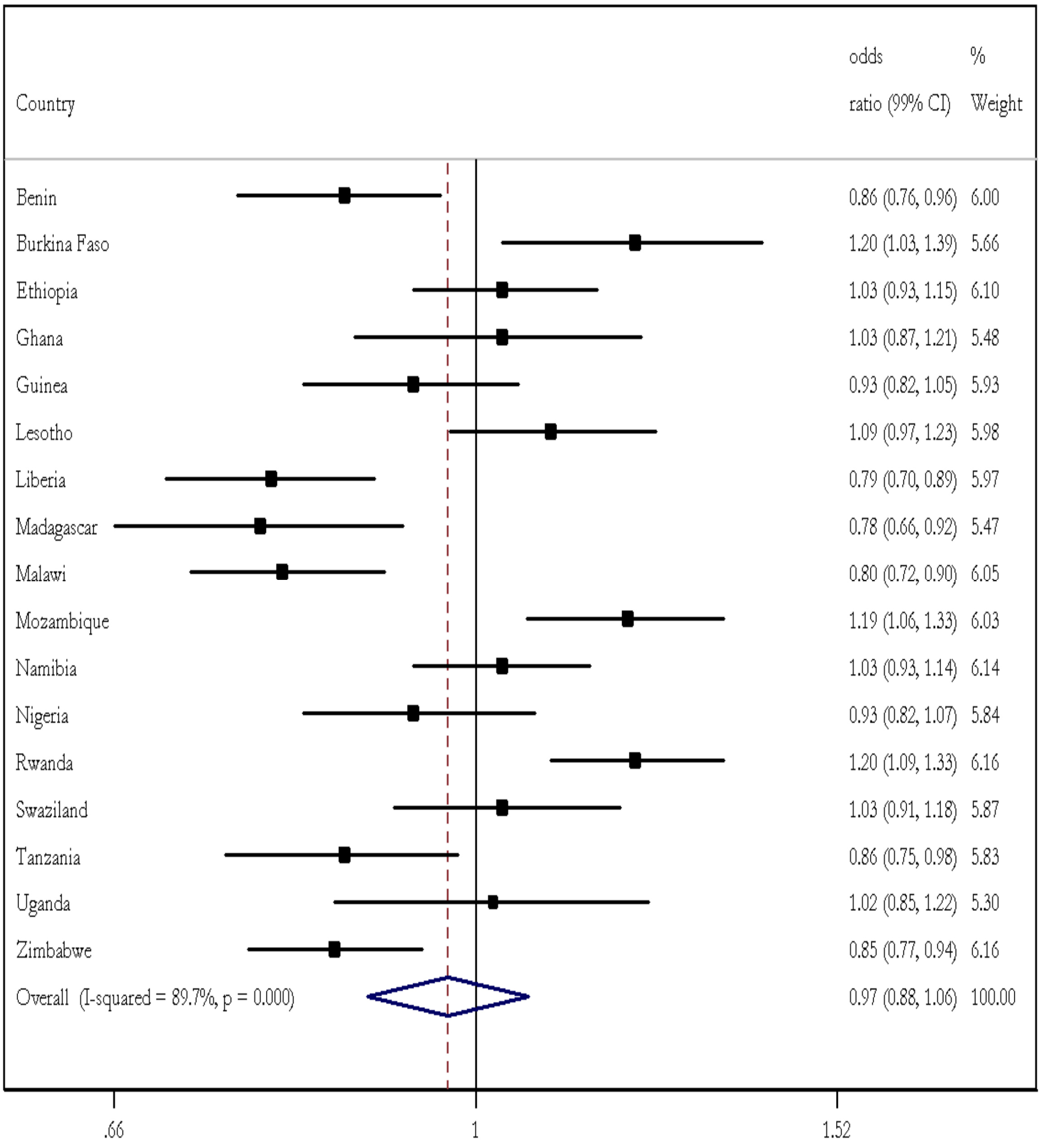

Forest plot of the odds ratios (ORs) and 99% confidence intervals (CIs) of individual countries and pooled data for currently married

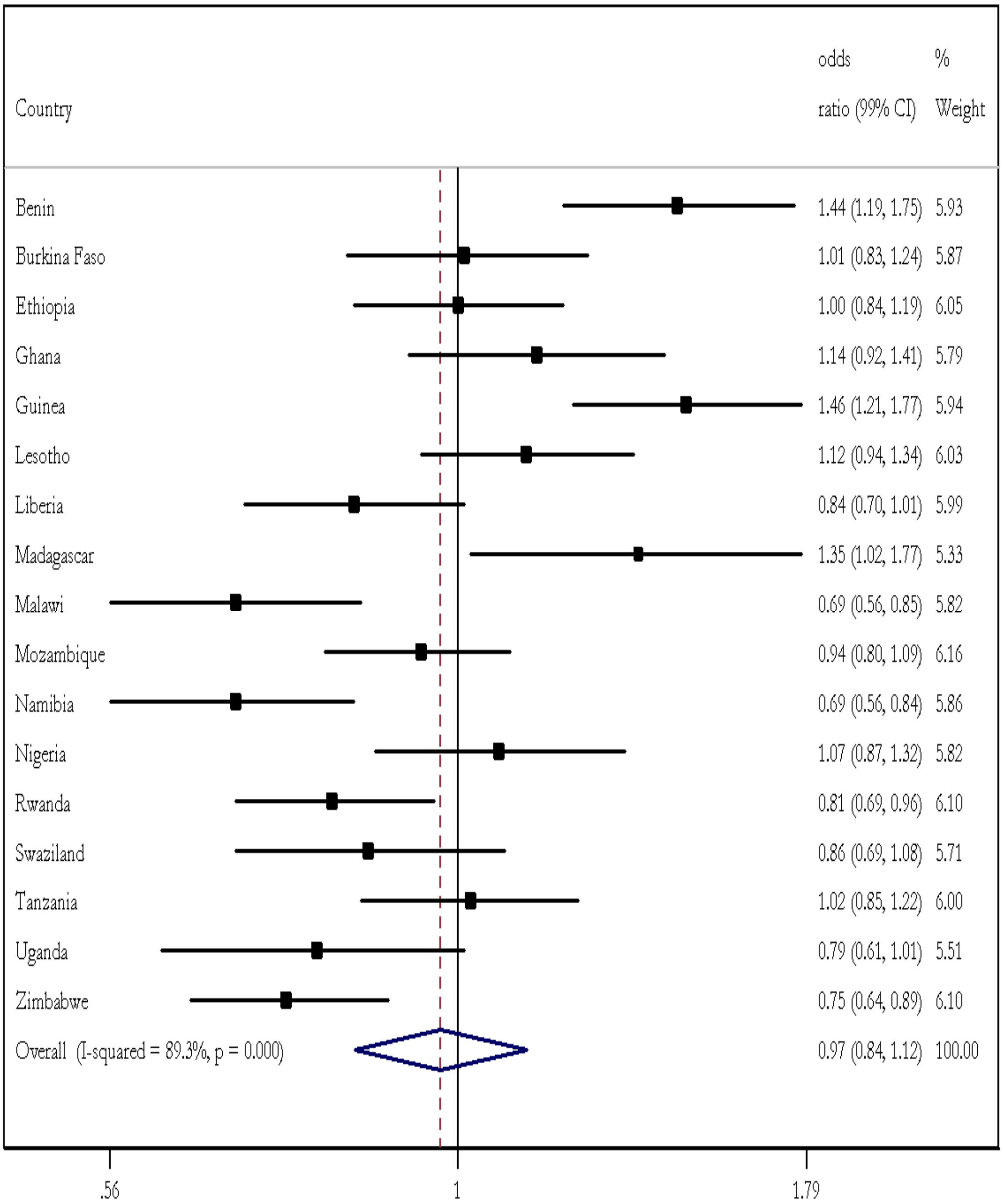

Forest plot of the odds ratios (ORs) and 99% confidence intervals (CIs) of individual countries and pooled data for formerly married

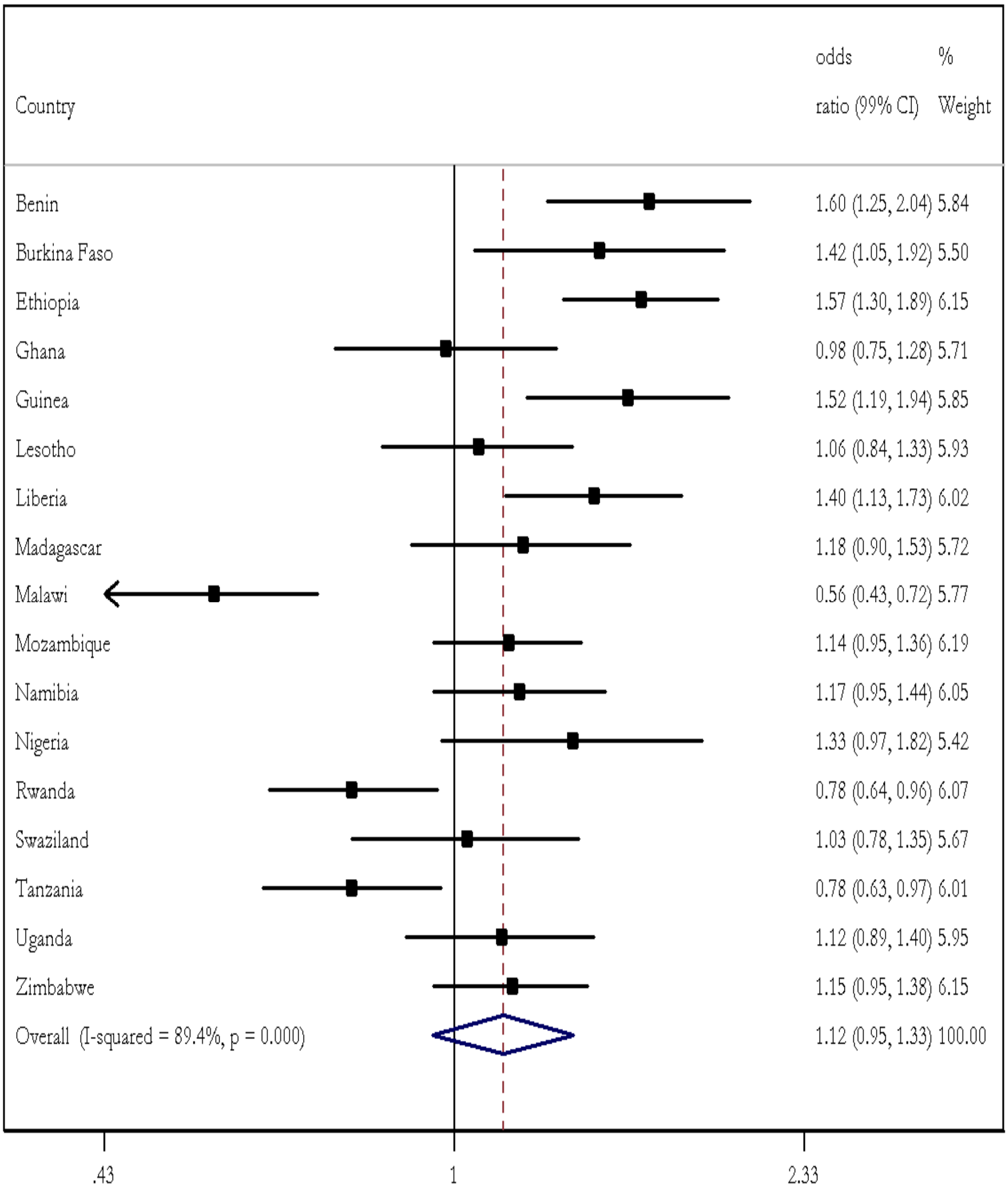

Forest plot of the odds ratios (ORs) and 99% confidence intervals (CIs) of individual countries and pooled data for wealth status (poorest)

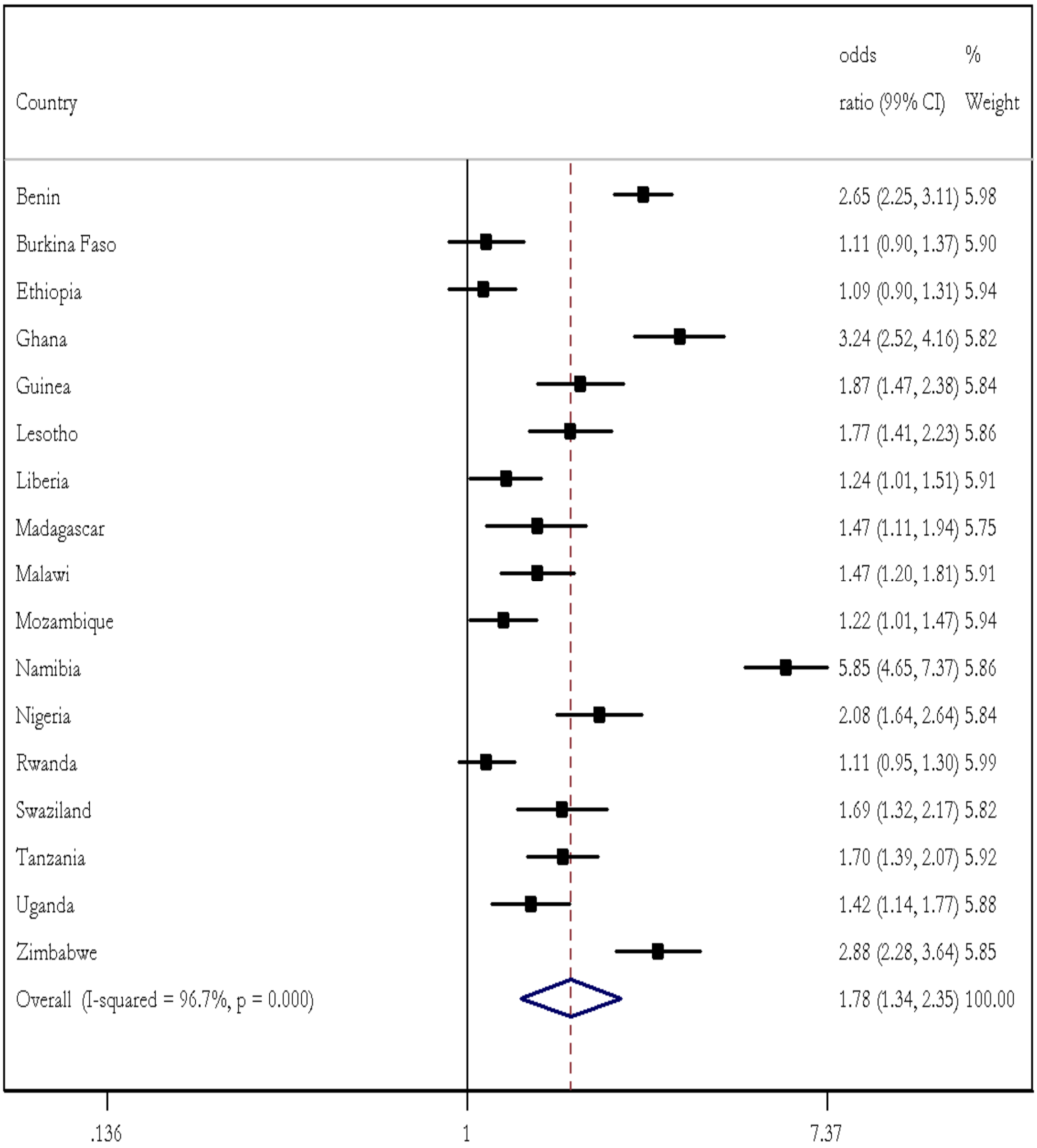

Forest plot of the odds ratios (ORs) and 99% confidence intervals (CIs) of individual countries and pooled data for wealth status (poor)

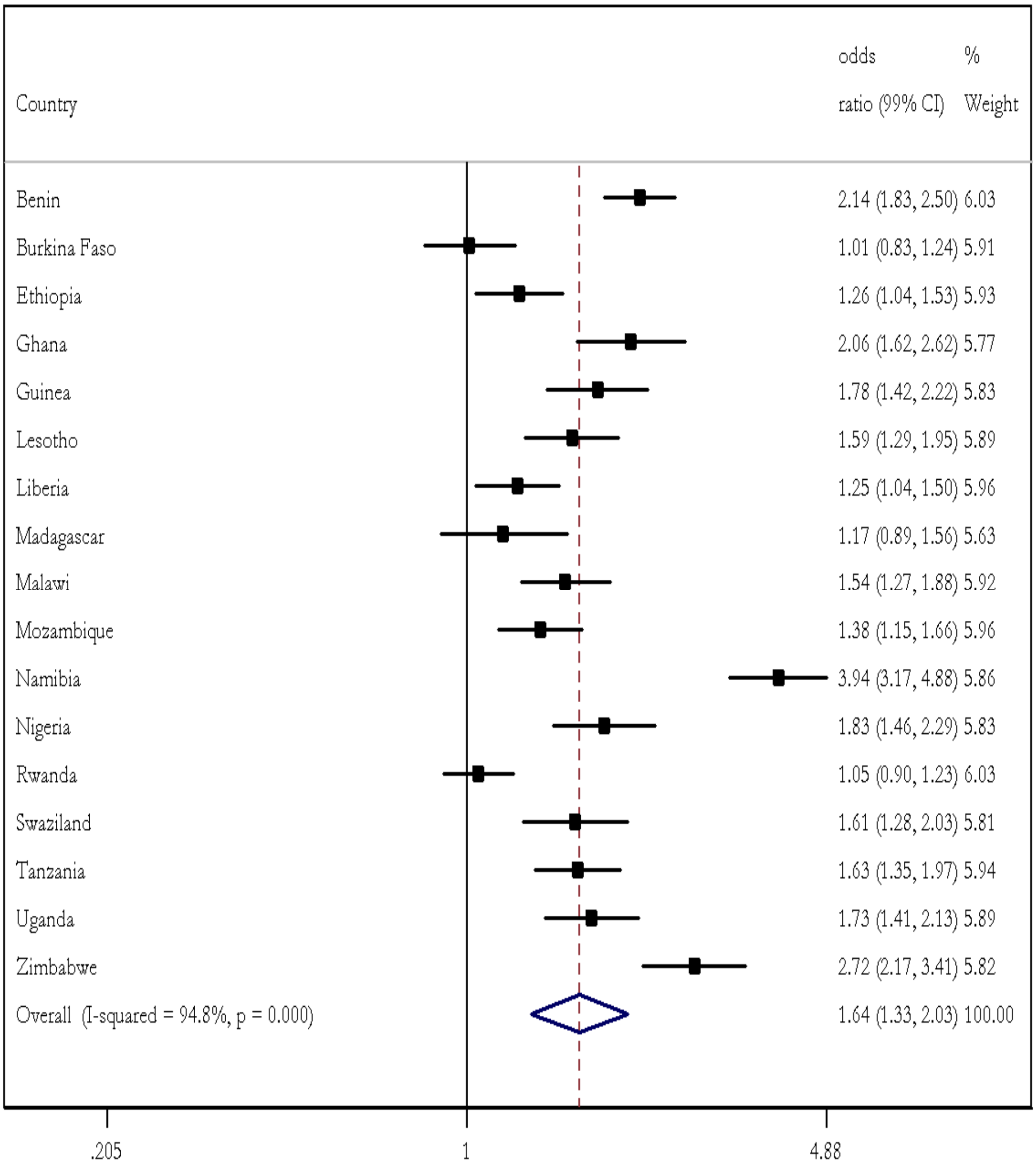

Forest plot of the odds ratios (ORs) and 99% confidence intervals (CIs) of individual countries and pooled data for wealth index (middle)

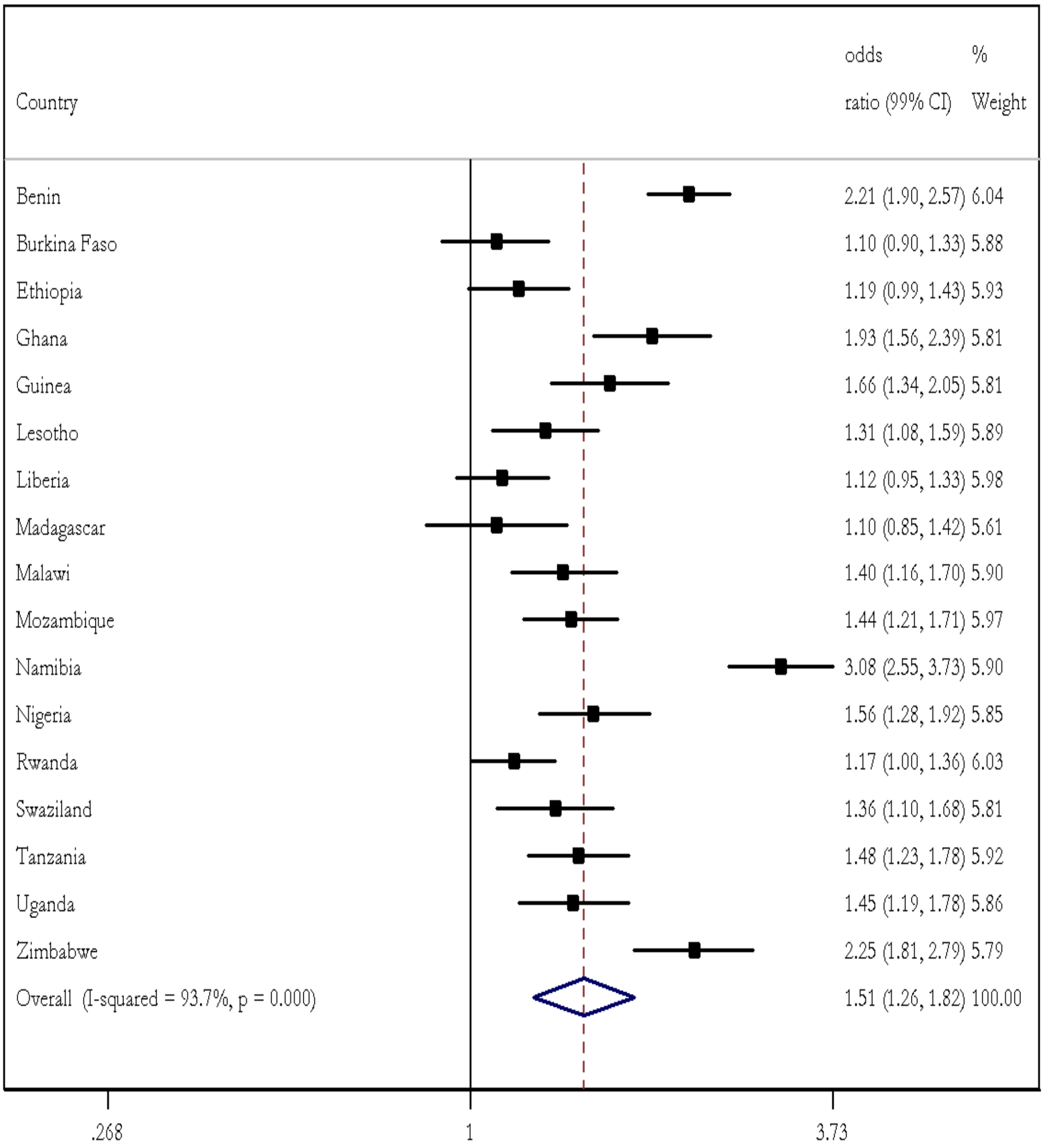

Forest plot of the odds ratios (ORs) and 99% confidence intervals (CIs) of individual countries and pooled data for wealth status (richer)

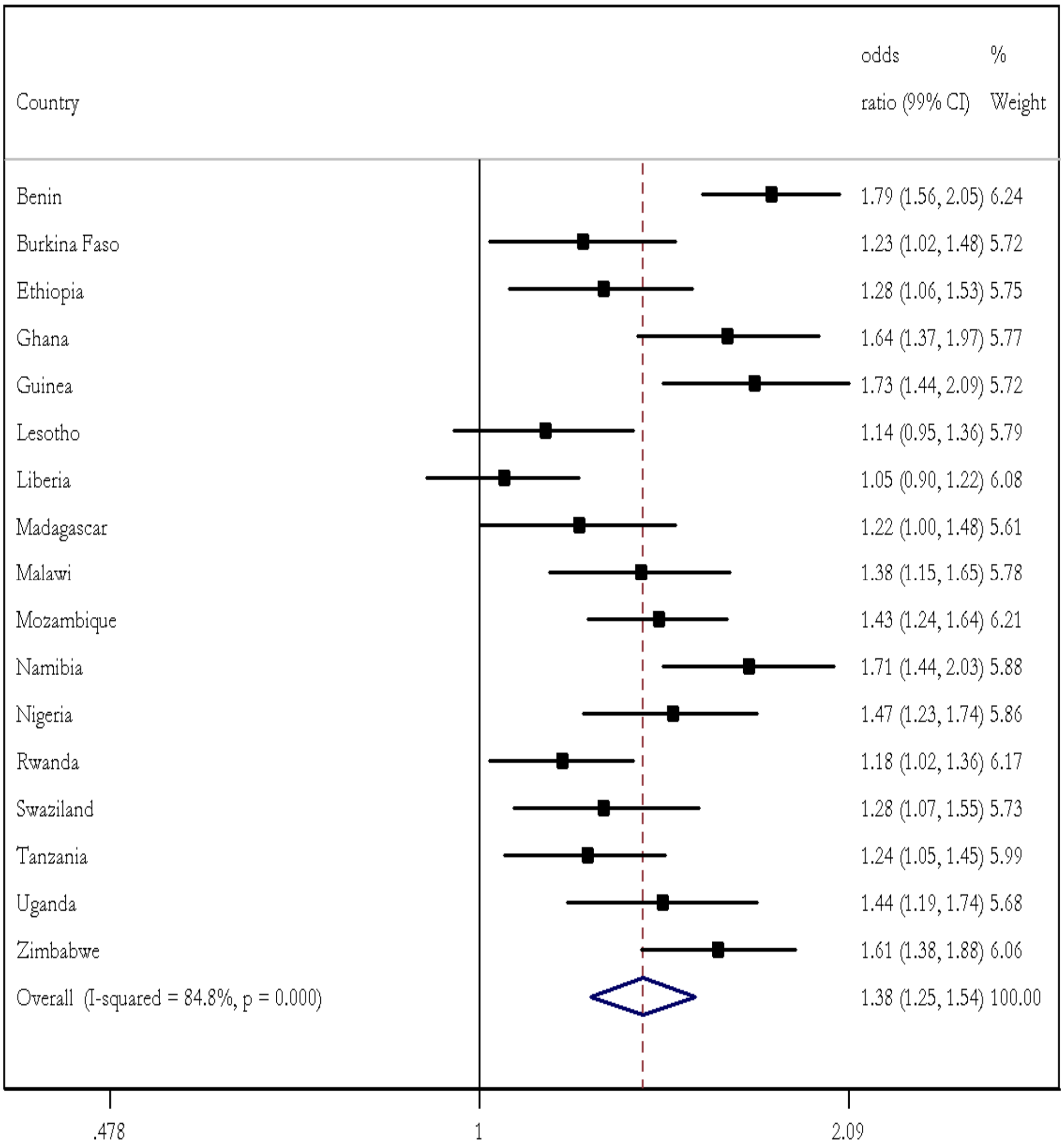

Forest plot of the odds ratios (ORs) and 99% confidence intervals (CIs) of individual countries and pooled data for place of residence

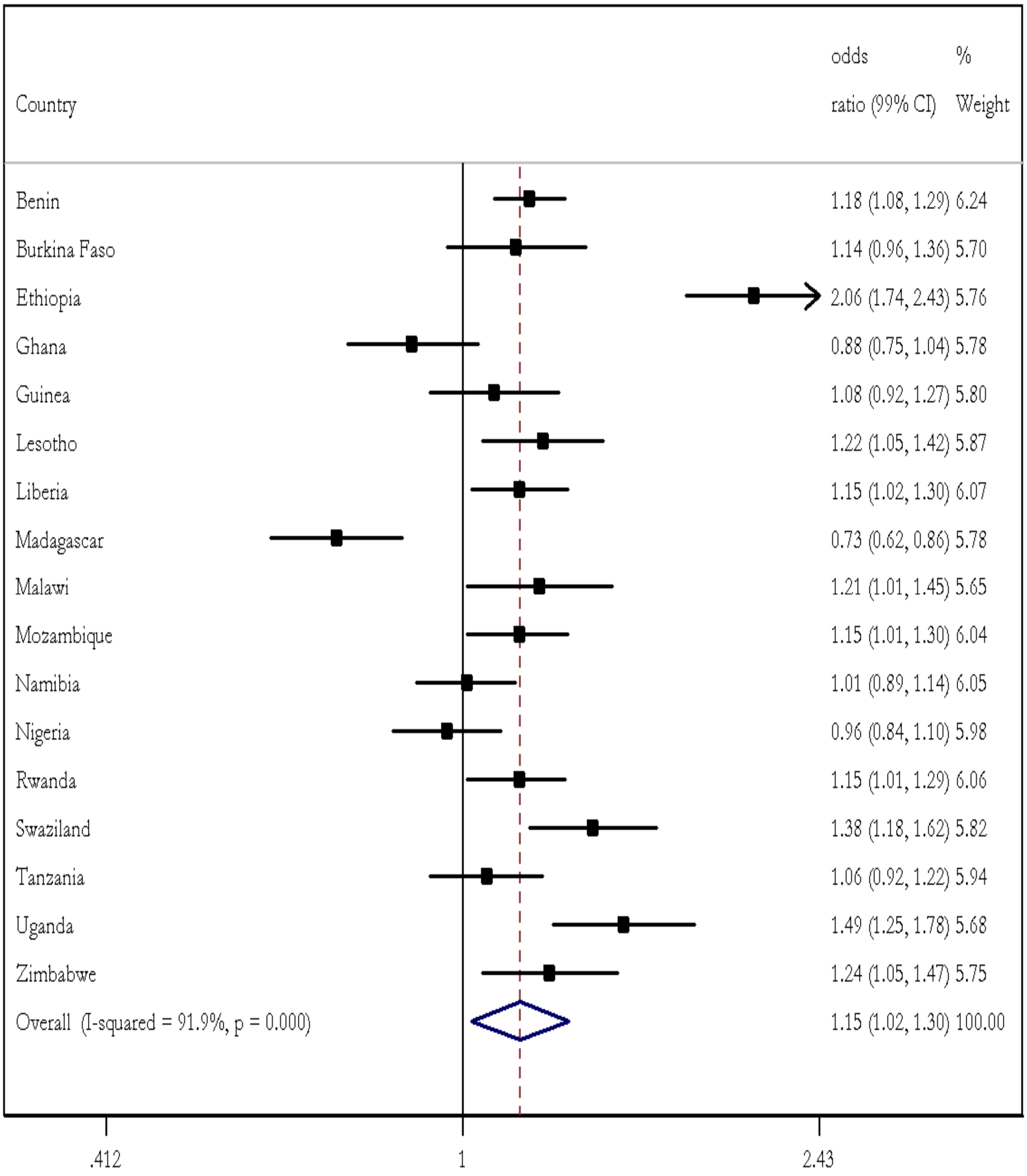

Forest plot of the odds ratios (ORs) and 99% confidence intervals (CIs) of individual countries and pooled data for decision making autonomy (respondent alone)

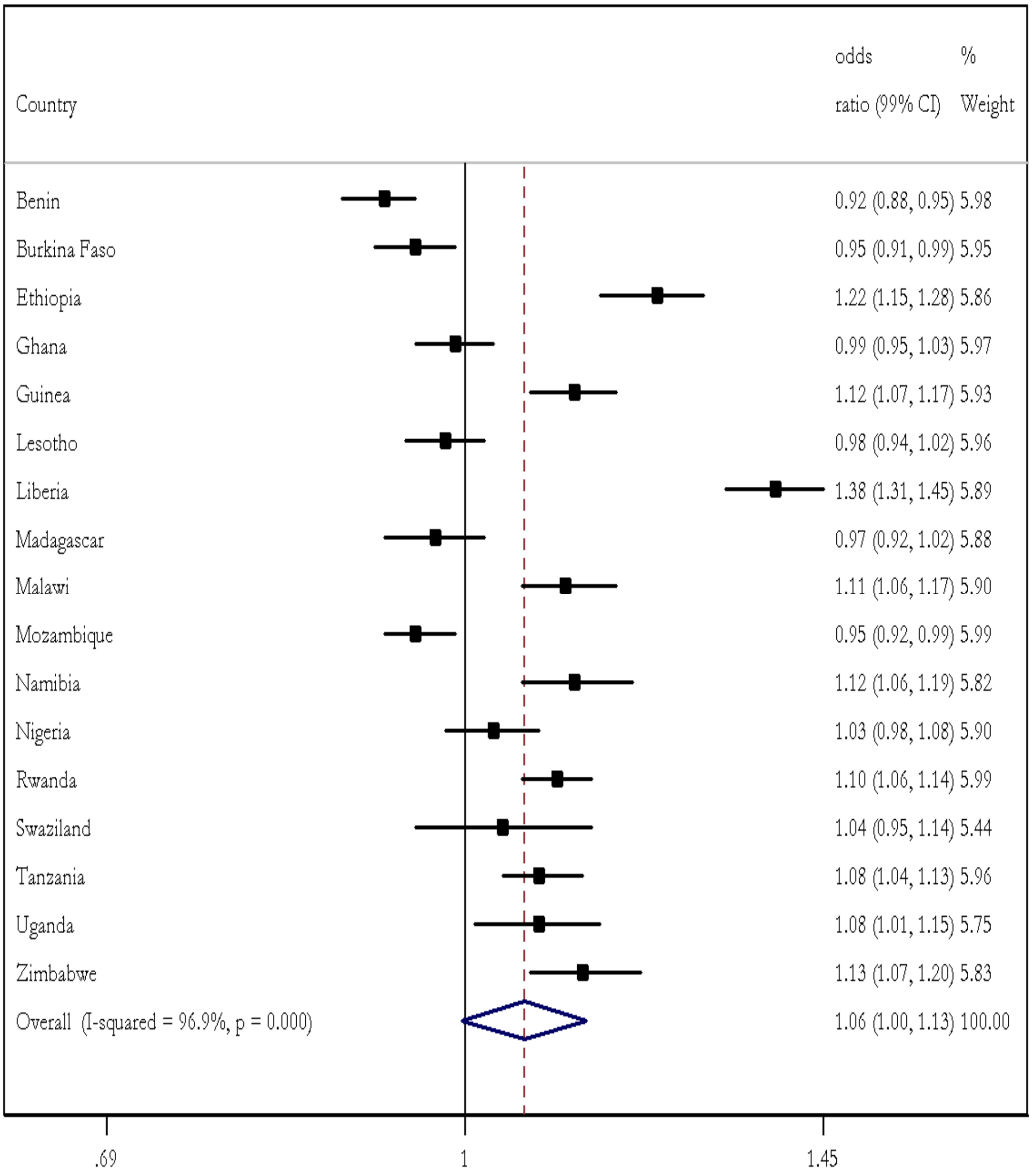

Forest plot of the odds ratios (ORs) and 99% confidence intervals (CIs) of individual countries and pooled data decision making autonomy (Husband/partner alone)

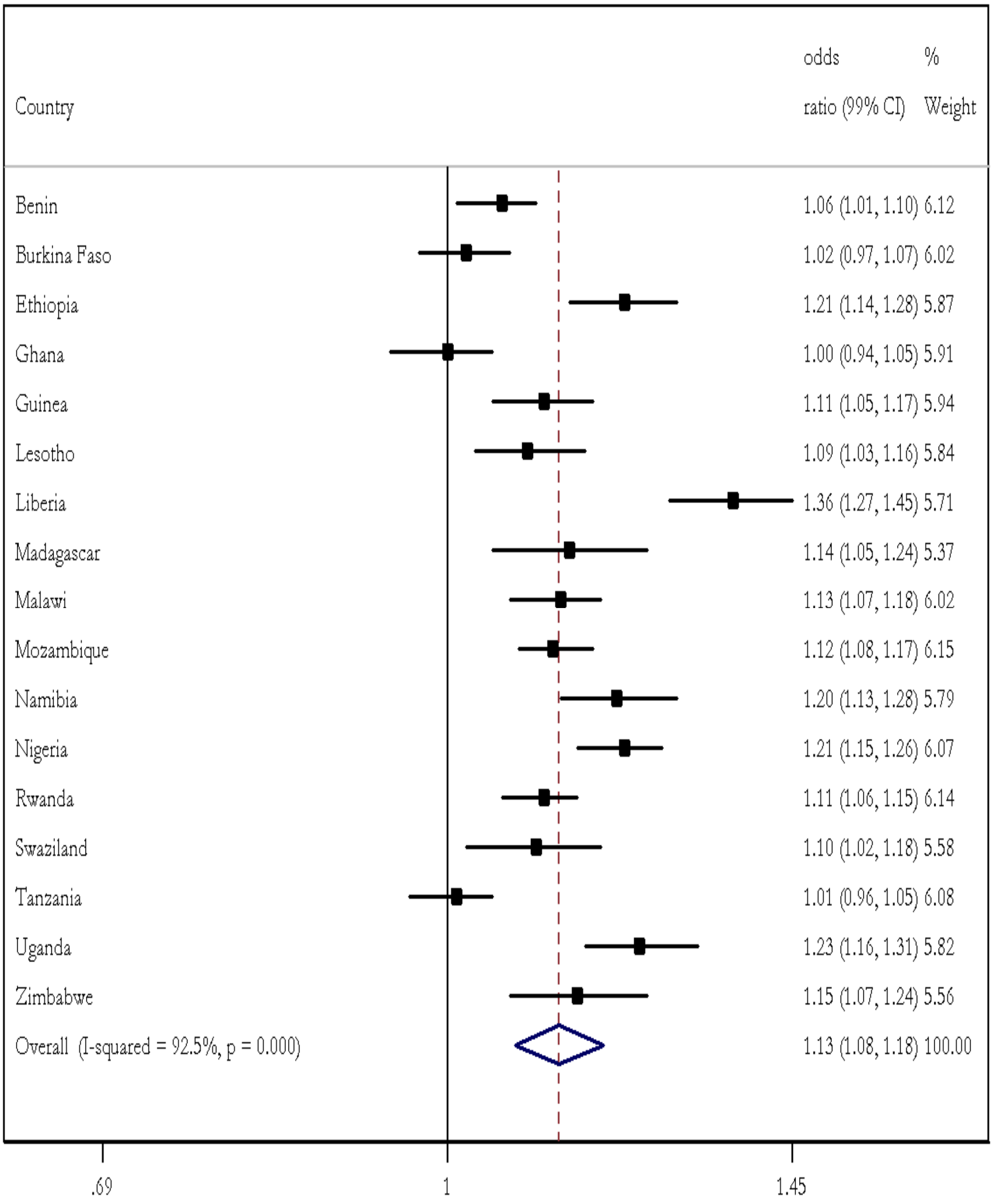

Forest plot of the odds ratios (ORs) and 99% confidence intervals (CIs) of individual countries and pooled data for decision making autonomy (Husband and wife)

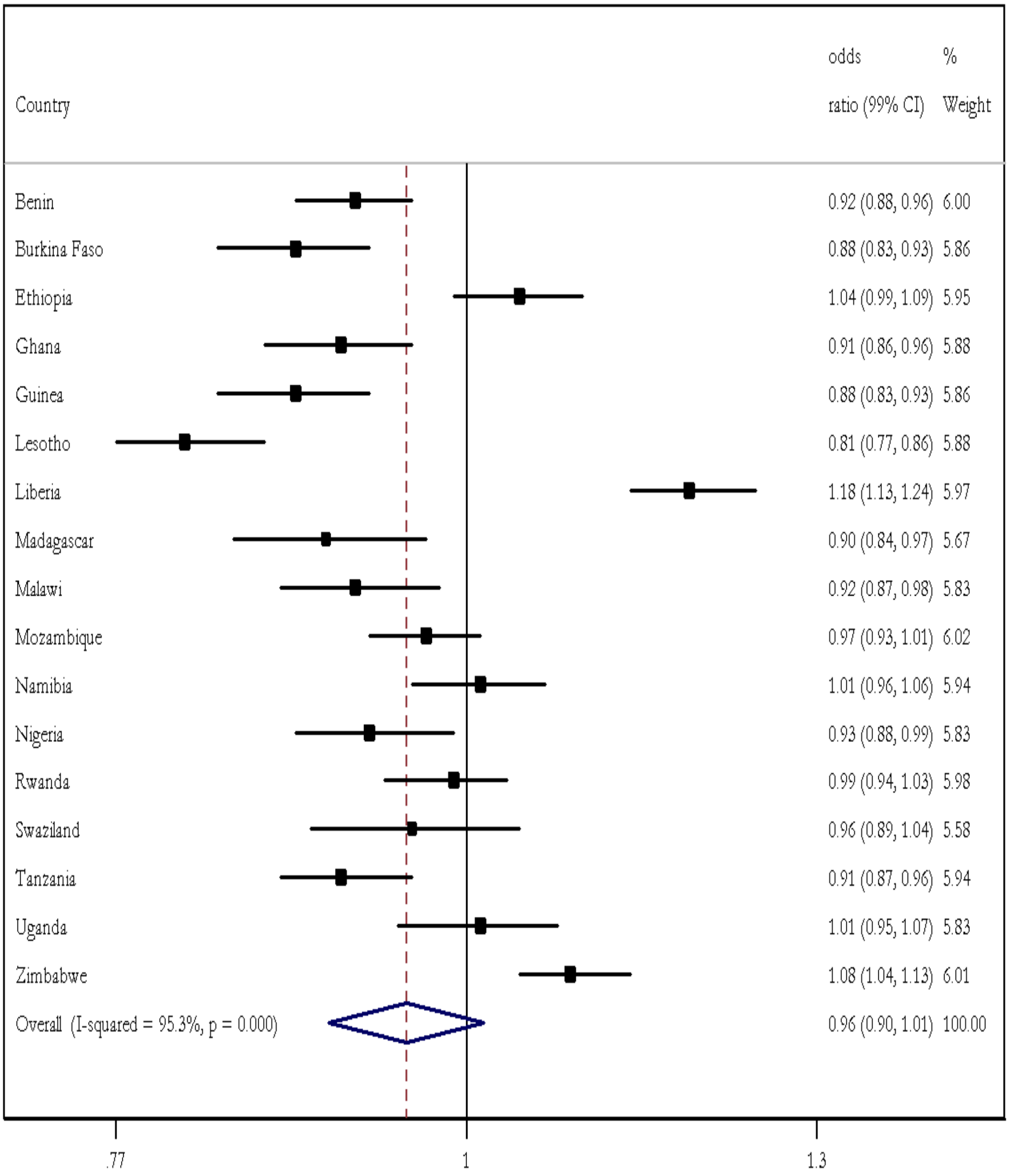

Forest plot of the odds ratios (ORs) and 99% confidence intervals (CIs) of individual countries and pooled data for access to newspaper

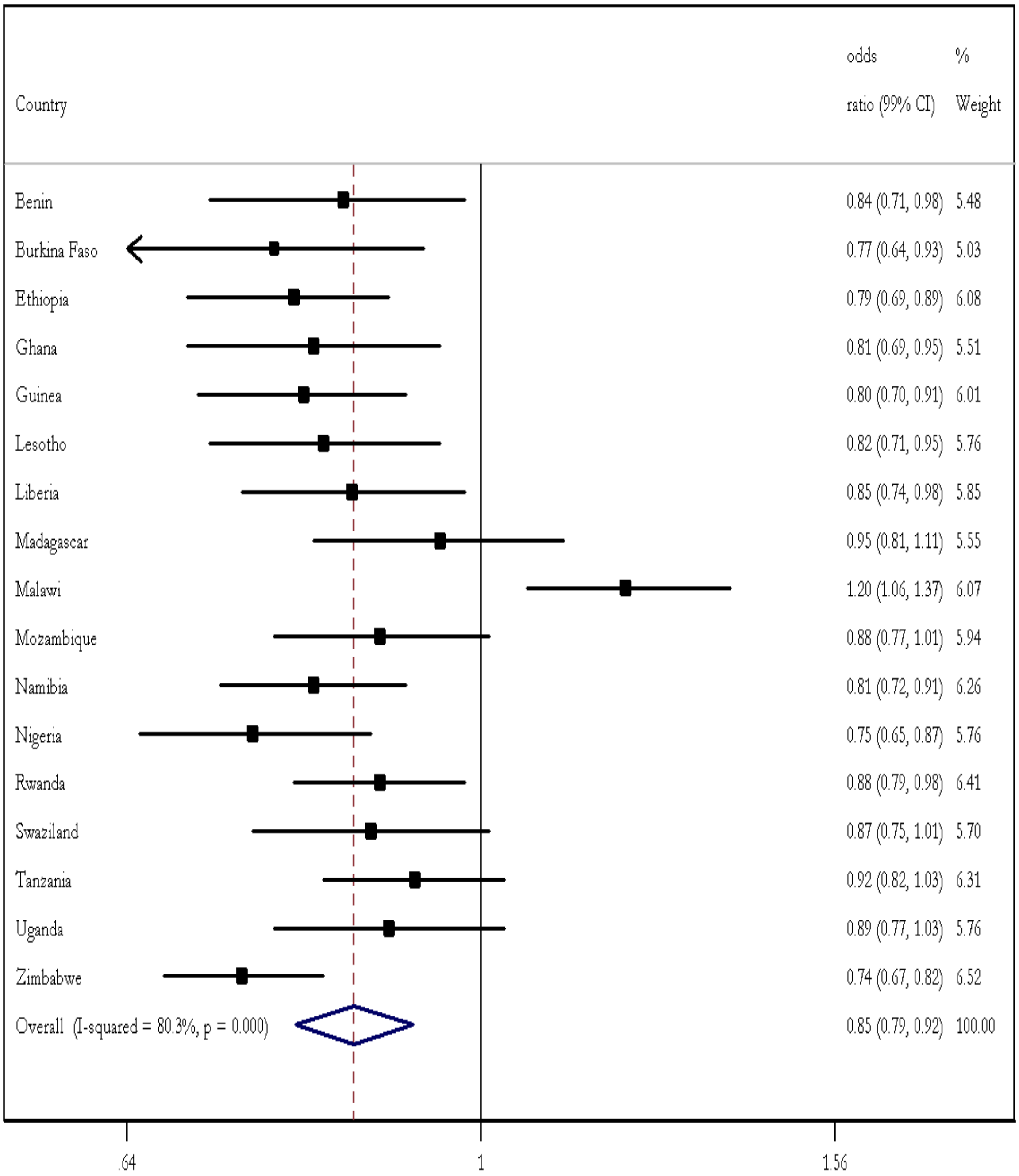

Forest plot of the odds ratios (ORs) and 99% confidence intervals (CIs) of individual countries and pooled data for access to radio

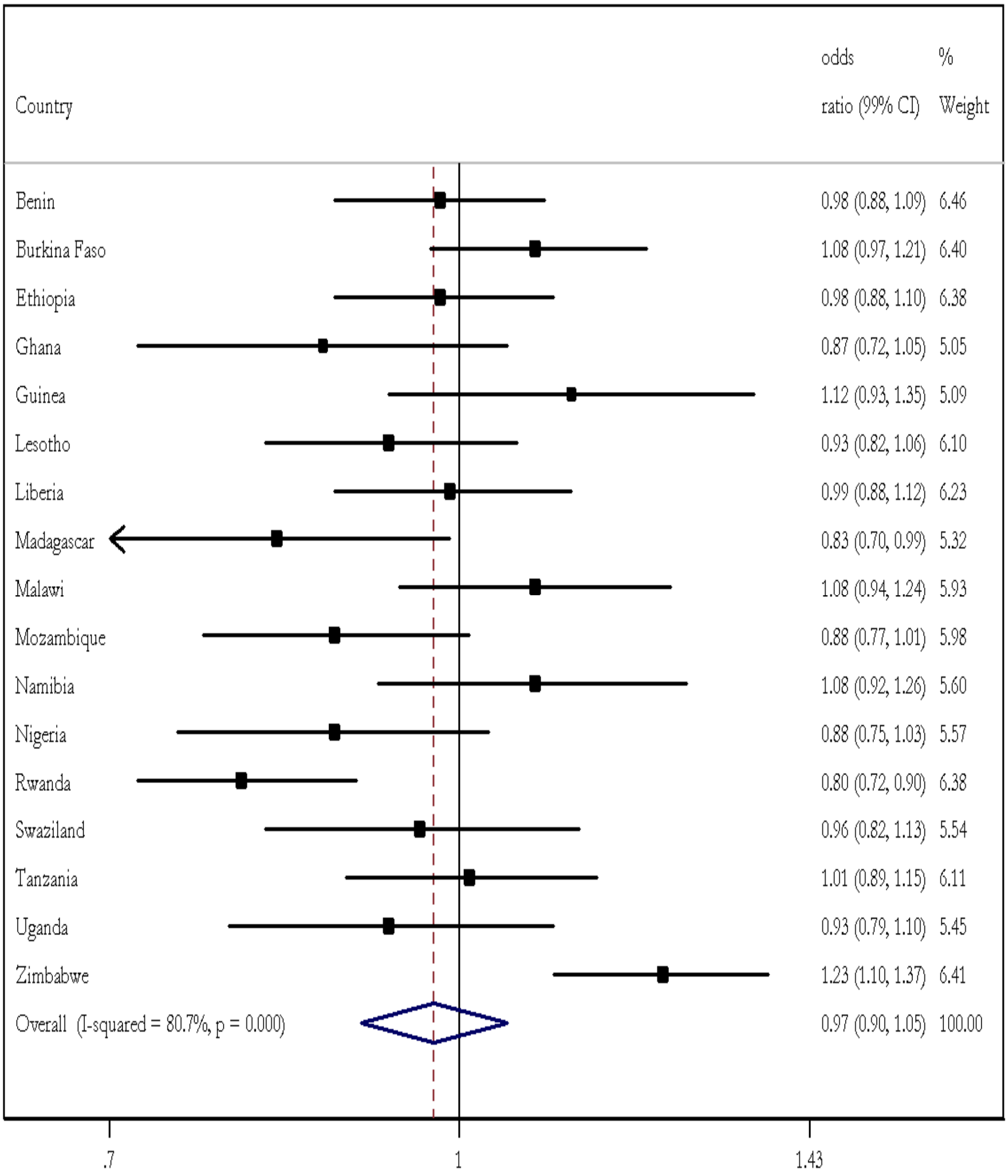

Forest plot of the odds ratios (ORs) and 99% confidence intervals (CIs) of individual countries and pooled data for access to television

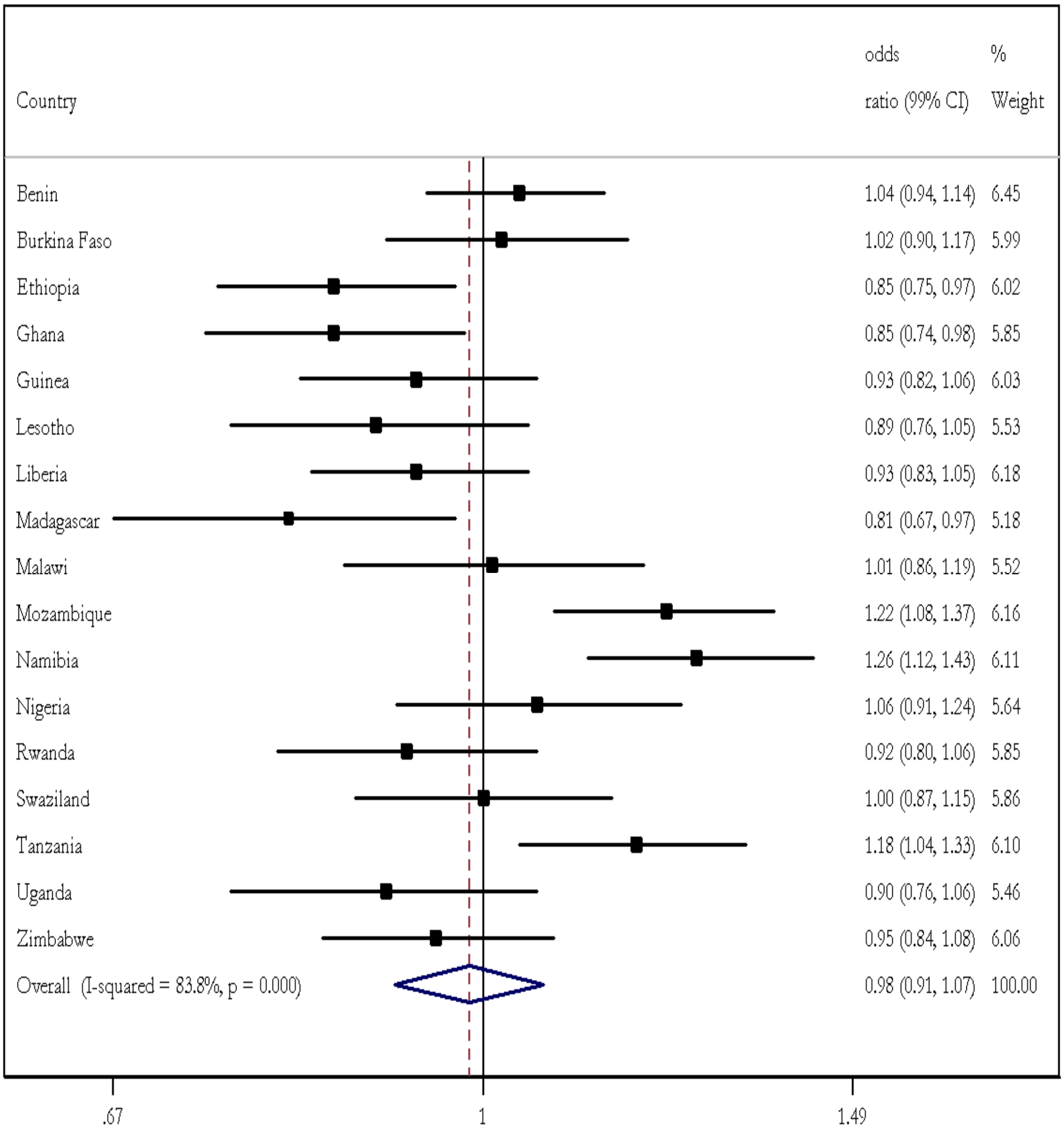

Supplement: Additional file 2 — Forest plot of the odds ratios (ORs) and 99% confidence intervals (CIs) of individual countries and pooled data for socio-demographic factors. [file 1472-698X-9-14-S2.pdf]
